# Insights into Convergent Evolution From Studying Amino Acid Patterns in Independent Lineages of Birds

**DOI:** 10.1093/gbe/evaf112

**Published:** 2025-12-15

**Authors:** Chul Lee, Seoae Cho, Kyu-Won Kim, Dongahn Yoo, Matthew Davenport, Jae Yong Han, Hong Jo Lee, Gregory Gedman, Jean-Nicolas Audet, Erina Hara, Miriam Rivas, Osceola Whitney, Andreas R Pfenning, Heebal Kim, Erich D Jarvis

**Affiliations:** Interdisciplinary Program in Bioinformatics, Seoul National University, Seoul, Republic of Korea; Laboratory of Neurogenetics of Language, The Rockefeller University, New York City, NY 10065, USA; eGenome, Inc., Seoul, Republic of Korea; Research Institute of Agriculture and Life Sciences, Seoul National University, Seoul, Republic of Korea; Interdisciplinary Program in Bioinformatics, Seoul National University, Seoul, Republic of Korea; Laboratory of Neurogenetics of Language, The Rockefeller University, New York City, NY 10065, USA; Department of Agricultural Biotechnology and Research Institute for Agriculture and Life Sciences, Seoul National University, Seoul, Republic of Korea; Department of Agricultural Biotechnology and Research Institute for Agriculture and Life Sciences, Seoul National University, Seoul, Republic of Korea; Division of Animal Sciences, University of Missouri, Columbia, MO, USA; Laboratory of Neurogenetics of Language, The Rockefeller University, New York City, NY 10065, USA; Laboratory of Neurogenetics of Language, The Rockefeller University, New York City, NY 10065, USA; Institute for Genomic Medicine, Columbia University College of Physicians and Surgeons, New York, NY 10032, USA; Integrated Laboratory Systems, Inc., Research Triangle Park, NC 27706, USA; Department of Biology, Division of Science, The City College of New York, New York, NY 10031, USA; Computational Biology Department, The School of Computer Science, Carnegie Mellon University, Pittsburgh, PA 15213, USA; Neuroscience Institute, Carnegie Mellon University, Pittsburgh, PA 15213, USA; Interdisciplinary Program in Bioinformatics, Seoul National University, Seoul, Republic of Korea; Laboratory of Neurogenetics of Language, The Rockefeller University, New York City, NY 10065, USA; Research Institute of Agriculture and Life Sciences, Seoul National University, Seoul, Republic of Korea; Laboratory of Neurogenetics of Language, The Rockefeller University, New York City, NY 10065, USA; Howard Hughes Medical Institute, Chevy Chase, MD 20815, USA

**Keywords:** convergent evolution, vocal learning, convergent variant finder (ConVarFinder), convergent single amino acid variant (ConSAV), product of original branch (POB) lengths, positive selection, song nuclei, *B3GNT2*, dopamine receptor D1B (DRD5)

## Abstract

Vocal learning, the ability to imitate sounds, is a complex convergent trait crucial for spoken language and observed in a few independent lineages of mammals and birds. While convergences in gene expression have been found in vocal learning brain regions, amino acid convergences remain unclear. Here, we investigated whether avian vocal learning clades have amino acid convergences linked to their specialized trait. We developed a tool, Convergent Variant Finder, and applied it to an alignment of 48 species representing nearly all bird orders to identify convergent single amino acid variants among vocal learners and over 8,000 other polyphyletic species combinations. We discovered that the number of convergent variants was associated with the product of branch lengths of the most recent common ancestors of each species combination. The number of convergent variants in vocal learning clades did not exceed that of control species combinations. However, a subset of genes with vocal learner–specific convergent amino acid variants was enriched in the “learning” process, under positive selection, and significantly overlapped with gene sets for *FOXP2* targets, singing-induced regulation in vocal learning nuclei, and differentially expressed in vocal learning nuclei. Moreover, we confirmed that the majority of convergent patterns in vocal learners were in the genomes of 363 species densely sampled across the avian tree. We propose that amino acid and nucleotide convergence accumulates at a steady state, with the rate proportional to divergence time. Selection associated with convergent traits, such as vocal learning, then likely acts on a subset of these changes.

Significance Convergent evolution is a common principle of biological systems, with vocal learning being one such example, a complex behavior that is a critical component of speech in humans and learned song in birds. Here, we developed a new tool to detect amino acid convergences amongst different combinations of species, including vocal learners, and found that their convergent amino acid frequencies were correlated with the product of ancestral branch lengths. Although vocal learners fell within the linear range of most other species combinations, their amino acid convergences were in genes enriched in learning-related functions. Based on our findings, we suggest that there is a steady background rate of convergent amino acid changes among avian species that is influenced by their branch lengths, for which selection acts upon convergent traits.

## Introduction

Single amino acid variants (SAVs) are one of the potential drivers of evolution for various traits. For example, the Forkhead box P2 (*FOXP2*) transcription factor has two well-known human-specific SAVs, which might have been positively selected for functions related to spoken language (Enard et al. 2002; Scharff and Petri 2011). Mice humanized for the two SAVs of *FOXP2* showed more advanced learning abilities (Schreiweis et al. 2014) and alterations of cortico-basal ganglia circuits (Enard et al. 2009; Enard 2011; Reimers-Kipping et al. 2011), which play critical roles in spoken language (Jarvis 2019); mice containing a heterozygous missense mutation that causes speech syllable apraxia in humans also show syllable sequencing deficits (Castellucci et al. 2016; Chabout et al. 2016).

**Fig. 1.**
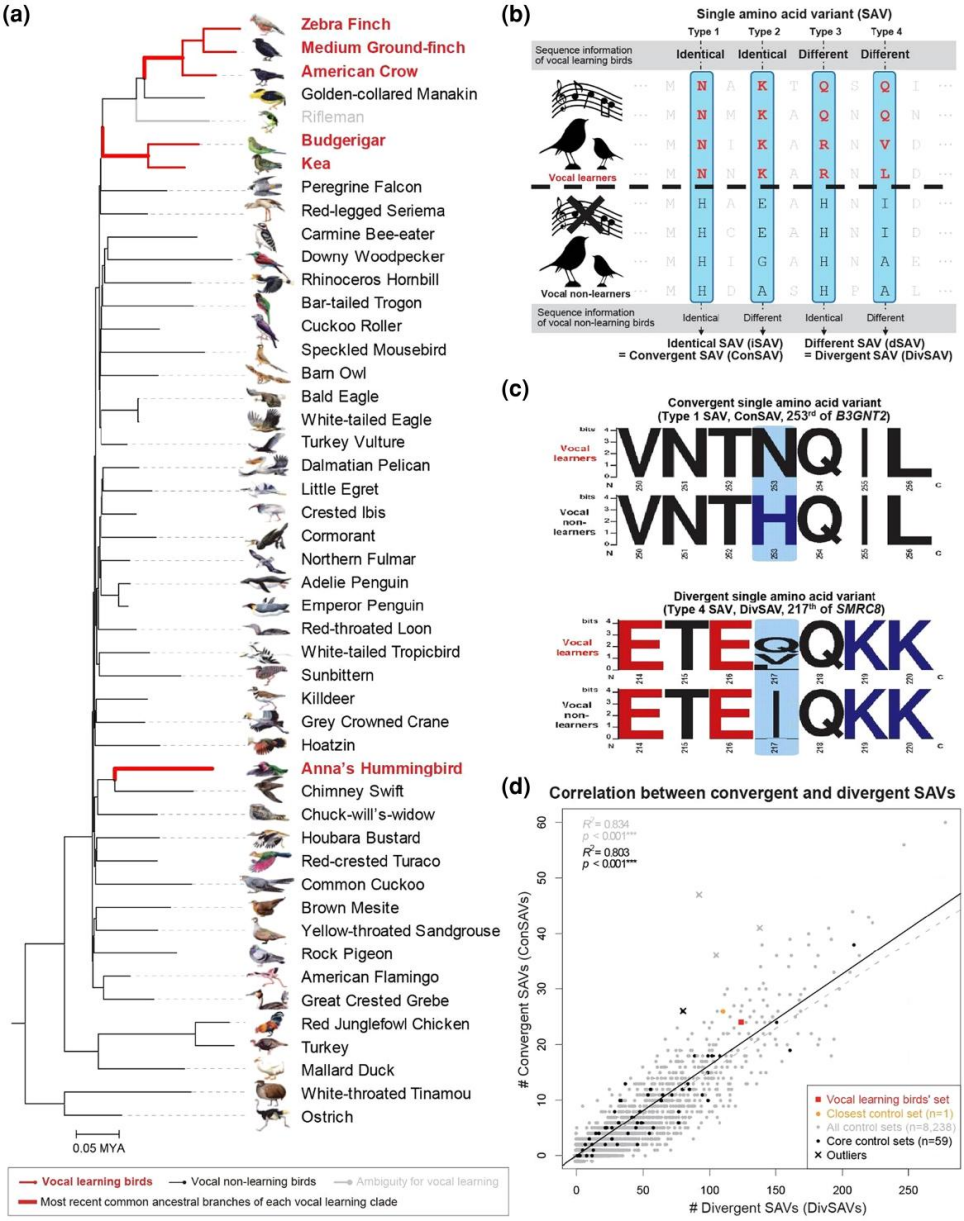
Convergent amino acid variants in vocal learning birds and other species combinations. a) Avian family tree and genomes analyzed. The branch lengths are estimated from the RAxML tree (Jarvis et al. 2014). Red bold text, vocal learning lineages. Bold red lines, most recent common ancestral branches (origin branch) of each vocal learning clade. b) Example illustration of the four types of convergent SAVs (sky blue–colored boxes), in vocal learning birds versus vocal nonlearning birds. c) Example amino acid sequence logos of a Type 1 ConSAV site in *B3GNT2* and a Type 4 DivSAV site in *SMRC8*. d) Correlation plots between amino acid convergences (ConSAVs; *y*-axis) and divergences (DivSAV; *x*-axis) of control species combinations consisting of 6 species from 3 independent lineages relative to 42 other species. Colors denote different types of species combinations, x = outliers. Two correlations are shown, for the core control set of 59 species combinations (black) and the broader control set of 8,238 species combinations (gray).

A crucial component of spoken language is vocal learning, the ability to produce imitated vocalizations. Advanced vocal learning is a convergent trait observed in only a few lineages of animals, including songbirds, parrots, and hummingbirds among birds (Fig. 1a), and bats, dolphins/whales, seals, elephants, and humans among mammals (Nottebohm 1972 ; Jarvis 2004; Petkov and Jarvis 2012; Nowicki and Searcy 2014; Jarvis 2019; Cahill et al. 2021). This is opposed to vocal nonlearners, who engage in vocalizations but do not imitate those made by others. Some studies have argued that vocal learning should not be considered a binary trait, but a continuum among species (Arriaga et al. 2012; Arriaga and Jarvis 2013; Jarvis 2019; Vernes et al. 2021; Wirthlin et al. 2024). This would mean that some species among the classified vocal nonlearners actually have some level of vocal learning, such as several suboscines and one duck species (Kroodsma et al. 2013; Ten Cate 2021; Ten Cate and Fullagar 2021). But these species are thus far outliers from their lineage, with more limited vocal learning abilities consistent with the continuum hypothesis. Together, these studies indicate that convergence for a complex trait can happen within a continuum.

At a neurobiological level, both vocal learners and vocal nonlearners share a similar auditory pathway that controls auditory learning, but only the advanced vocal learning clades thus far examined (songbirds, parrots, hummingbirds, humans, and bats) have been found to share a specialized convergent forebrain pathway that controls vocal learning and production of learned vocalizations (Jarvis 2004; Petkov and Jarvis 2012; Nowicki and Searcy 2014; Pfenning et al. 2014; Wirthlin et al. 2024). Supporting the hypothesis of independent origins of vocal learning brain pathways, the first genome-scale phylogenetic trees of birds showed that the three avian vocal learner lineages are indeed not monophyletic (Jarvis et al. 2014; Feng et al. 2020). Even though songbirds and parrots are relatively closely related, their two closest relatives, suboscines and New Zealand wrens (e.g. Rifleman), are considered predominantly vocal nonlearning lineages based on their innate vocalizations without auditory experiences (Touchton et al. 2014), except for Cotingidae bellbirds (Kroodsma et al. 2013).

In the first genome-scale analyses for vocal learning among birds, genes with positively selected changes in zebra finch (a songbird) compared with chicken (a Galliformes) were identified (Warren et al. 2010). Some of the positively selected genes were ion channels, which are known to control neurological function, behavior and disease (Warren et al. 2010). However, the comparison by necessity at that time was narrow, between only one vocal learner (zebra finch) and one vocal nonlearner (chicken), which are also very distant relatives (Jarvis et al. 2014) with a similar divergence level between marsupial and placental mammals.

The first draft genome sequences of the Avian Phylogenomics Project (Bird 10K project, B10K) consisted of 48 avian species representing nearly all bird orders (Zhang et al. 2014), which provided an unprecedented opportunity to investigate genetic features specific to polyphyletic vocal learning clades. These studies found convergent brain gene expression specializations in vocal learning birds and human (Jarvis 2004; Petkov and Jarvis 2012; Pfenning et al. 2014). In preliminary analyses with these genomes, we also found mutually exclusive amino acid substitutions unique to vocal learners, using a novel method (target-specific amino acid substitutions [TAAS] analysis) (Zhang et al. 2014). However, the latter study had not considered emerging viewpoints reported around that time for principles of molecular convergence (Castoe et al. 2009; Foote et al. 2015; Goldstein et al. 2015; Thomas and Hahn 2015; Thomas et al. 2017); it did not separately test for convergent (identical) versus divergent (different) amino acid substitutions; it did not compare any control species combinations, with phylogeny similar to vocal learning birds; and it did not test for possible influences of close relatives and different numbers of species in each lineage.

Here, we overcame the above limitations. We developed a new computational method, Convergent Variant Finder (ConVarFinder), to identify convergent and divergent amino acid substitutions unique to vocal learning lineages or any other polyphyletic species combination, by considering phylogenetic relationships and estimating ancestral sequences. Control sets of different species combinations and their molecular convergences and divergences were compared with that of vocal learning birds. Based on these comparisons, we discovered that the proportion of genetic convergences were dependent on phylogenetic properties of polyphyletic combinations of species. Despite avian vocal learning clades not having a higher level of convergence than expected, multiple lines of evidence for a subset of the candidate genes with convergent amino acids in vocal learning birds indicated associations with learning functions. These findings have implications for general principles of convergent evolution.

## Results

### Convergent Amino Acid Variants Specific to Avian Vocal Learning Clades

Based on the previous TAAS algorithm (Zhang et al. 2014), we developed a new tool called ConVarFinder (Fig. S1; Materials and Methods), which scans sequence alignments for convergent amino acid and other types of variants that are mutually exclusive to a polyphyletic group of species A versus a group of species B (Fig. 1a). ConVarFinder classifies the mutually exclusive variants into the following four types (Fig. 1b): Type 1, mutually exclusive identical substitutions in species Group A and species Group B; Type 2, identical substitutions in Group A and different substitutions in Group B; Type 3, the inverse of Type 2, with different substitutions in Group A not shared with identical substitution in Group B; and Type 4, mutually exclusive different sets of substitutions between Groups A and B. We call the Types 1 and 2 with identical substitutions in species group A as convergent variants in that group and the Types 3 and 4 with different substitutions in species group A as divergent variants in that group. In this manner, the broad term “convergent” traditionally refers to all four types of variants, but the specific term *convergent* we use here applies just to Types 1 and 2 and *divergent* to Types 3 and 4. Compared with previous studies on convergent evolution in reptile and mammalian lineages that tested pair-wise combinations of two species at a time (Castoe et al. 2009; Goldstein et al. 2015; Thomas and Hahn 2015), our approach can test multiwise species combinations.

We applied ConVarFinder to the genomes of 48 avian species (Zhang et al. 2014) spanning most orders (Jarvis et al. 2014). We first scanned the alignments of 8,295 1:1 orthologous protein-coding genes consisting of 4,519,041 homologous amino acid sites for shared amino acid patterns in 6 vocal learning species from the 3 vocal learning orders or suborders (songbirds: zebra finch, medium ground finch, and American crow; parrots: budgerigar and kea; and hummingbirds: Anna's hummingbird) that were not found in any of the remaining 41 assumed vocal nonlearning species (Fig. 1a). Rifleman, a New Zealand wren and a close relative to vocal nonlearning suboscines and both in turn close relatives to vocal learning oscines, was included in the alignments but initially excluded in the convergent amino acid identifications because of the uncertainty of its vocal learning ability, although assumed to be a vocal nonlearner (Jarvis et al. 2014).

We found 148 sites (0.0033% of total sites) in 135 genes (1.6% of total genes) with SAVs specific to avian vocal learners (Table S1). Of these 148, 3 and 21 were classified as Type 1 and 2 convergent SAVs (ConSAVs; 24 total), respectively, and 6 and 118 were classified as Type 3 and 4 divergent SAVs (DivSAVs; 124 total), respectively. Logically, Type 1 was in the minority and Type 4 in the majority of substitutions because Type 1 has the most stringent criterion, requiring each group of species to have identical substitutions. In contrast, Type 4 had the most relaxed criterion, allowing multiple substitutions in each polyphyletic clade. An example of Type 1 was the 253rd site of *B3GNT2* with an asparagine (N) in all avian vocal learning species examined and a histidine (H) in all the remaining species examined; an example of Type 4 is the 217th site of *SMRC8* with a glutamine, valine, or leucine (Q, V, or L) in all avian vocal learners, and alanine (A) or isoleucine (I) in all other species (Fig. 1c).

Within our ConVarFinder toolbox (Fig. S1), we performed ancestral sequence reconstruction on the variants using RAxML (Stamatakis 2014) and the avian family tree in Fig. 1a (Jarvis et al. 2014). We confirmed that all 148 avian vocal learner–specific SAV sites had inferred evolutionary directions of either convergent identical or divergent different substitutions in vocal learners relative to their most recent common ancestors (MRCAs; Table S1). For the 24 avian vocal learner–specific ConSAVs, 1 took a simple parallel path from an identical amino acid at ancestral nodes to another identical amino acid in only vocal learners; the remaining 23 took complex convergent paths with intermediate substitutions of different amino acids at ancestral nodes to finally an identical amino acid in only vocal learners. Of the 124 avian vocal learner–specific DivSAVs, 4 took a simple path and 120 took complex divergent evolutionary paths. These findings indicate that ConVarFinder is a robust toolbox for identifying and classifying various types of amino acid convergences in a group of species, given a tree, and that vocal learning bird species have several or more of all types.

### Convergent Versus Divergent Variant Relationships Across Multispecies Combinations

We next tested whether avian vocal learners have a higher frequency of convergent substitutions relative to control sets of species. Considering the polyphyletic relationship of the 6 vocal learning species examined, we designed 2 types of clade-specific control sets based on 10,737,573 possible species combinations (Fig. S2): (i) all controls consisting of 8,238 different polyphyletic species combinations given a phylogeny with 6 target species from 3 independent lineages, regardless of whether they included vocal learners or not and (ii) of these 8,238 control combinations, 59 core controls consisting of all possible combinations of 6 target species having at least 2 vocal learning clades and 1 nonlearning clade. For example, the latter included the control combination of songbirds and parrots as two vocal learning clades and swift as a vocal nonlearning clade, a close relative to hummingbirds. We conducted ConVarFinder analyses on each of the 8,238 control species combinations and identified their amino acid convergences and divergences.

We found a strong correlation between the number of ConSAVs and DivSAVs across species combinations (Fig. 1d). There were four outlier species combinations (x in Fig. 1d), all with relatively higher numbers of ConSAVs to DivSAVs than expected given the regression line (adjusted *P* > 0.05); but none of them were the vocal learner group. Among the outliers, the highest residual was 32.46 in a combination that included 4 passerines (songbirds and a suboscine), a parrot, and a falcon, and 17.61 in a core control that included 3 songbirds, Anna's hummingbird, and 2 land fowls (Fig. 1d). These outlier species combinations do not share known convergent traits as far as we are aware. These findings indicate that amino acid convergences are widespread for birds, and the numbers of convergent and divergent variants vary in a linear fashion for different species combinations.

### Product of Most Recent Ancestor Divergence Times Influences Amino Acid Convergences

**Fig. 2.**
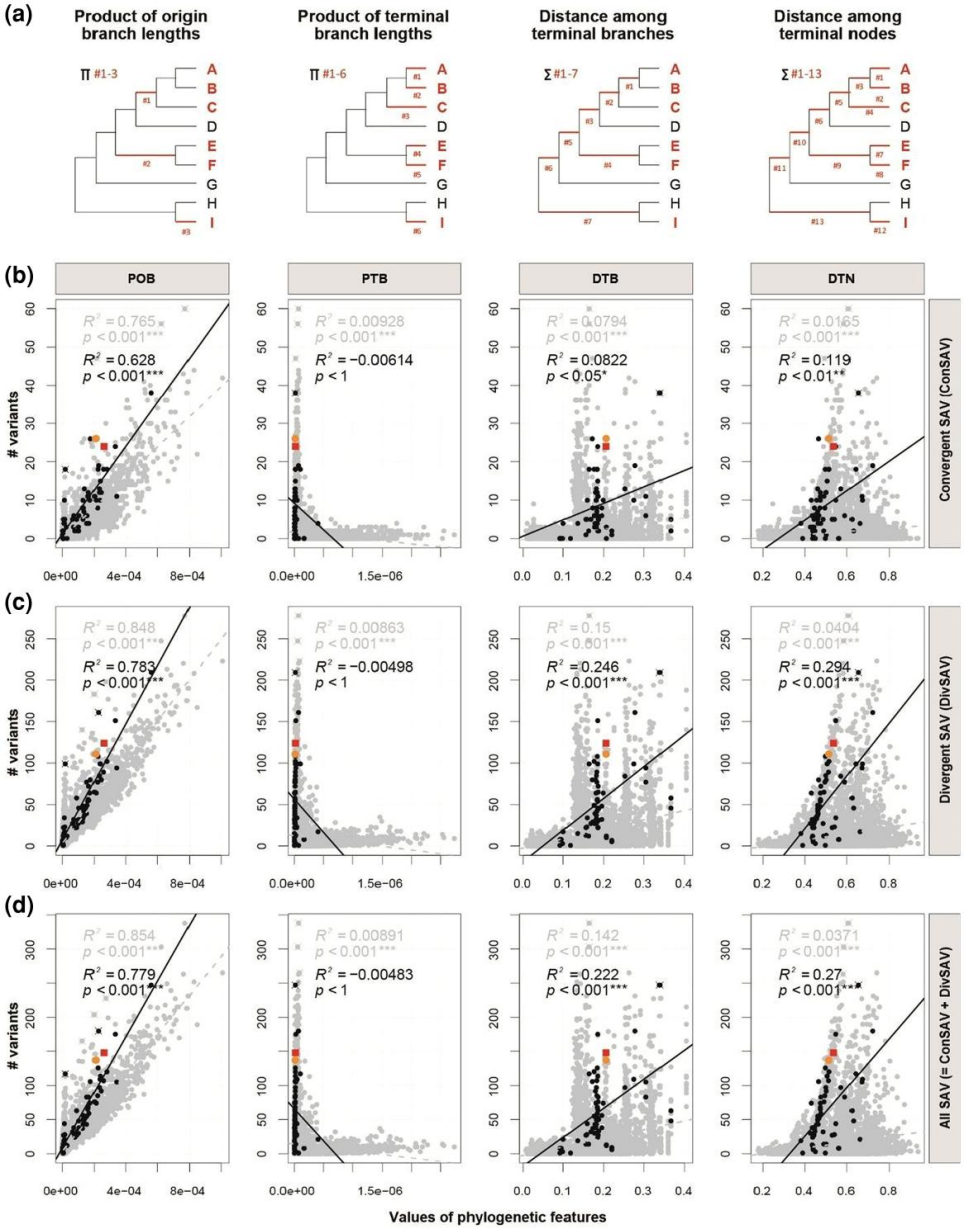
Phylogenetic features correlated with amino acid convergences. a) Four types of phylogenetic tree features measured: POB lengths, PTB lengths, DTBs, and DTNs. In the example trees, red bold lines show the branches used for the calculations and red texts show the species clades that have a convergent trait. b to d) Regression analyses of each type of phylogenetic feature (columns) with three categories for SAVs (rows): ConSAVs, DivSAVs, and total convergent and divergent SAVs (ConSAVs + DivSAVs, All SAVs) in all species combinations examined, the vocal learning set and control sets of avian species. Color coding is the same as in Fig. 1d.

According to previous studies on mammalian and drosophila nuclear genomes (Zou and Zhang 2015) and vertebrate mitochondrial genomes (Goldstein et al. 2015), fewer convergent substitutions are expected with greater phylogenetic branch distances reflecting time to accumulate mutations. However, the correlations found in those studies showed high levels of variation, which make it difficult to identify principles reliably as well as identify the outliers. Here, we analyzed these and many other phylogenetic variables (Fig. 2a). We found strong and significant correlations for both ConSAVs and DivSAVs with the product of the MRCA origin branch (POB; Fig. 2b to d) lengths. This was the case for both the broad control set and core control set of species combinations, with a steeper slope for the former (Fig. 2b to d). Much weaker to no correlations were observed with the product of terminal branches (PTB), distances among terminal branches (DTBs), and distances among terminal nodes (DTNs; Fig. 2b to d). Like in the ConSAV versus DivSAV correlation analyses (Fig. 1d), although there were several outliers (marked by x) in multiple analyses, the avian vocal learners were not among them (Fig. 2). These findings suggest that POB largely explains the proportions of various types of amino acid substitutions in polyphyletic species combinations, where the longer evolutionary time on the ancestral branch of the polyphyletic species combination the greater frequency of convergent substitutions.

### Convergent Nucleotide Change Patterns That Explain Convergent Amino Acids

**Fig. 3.**
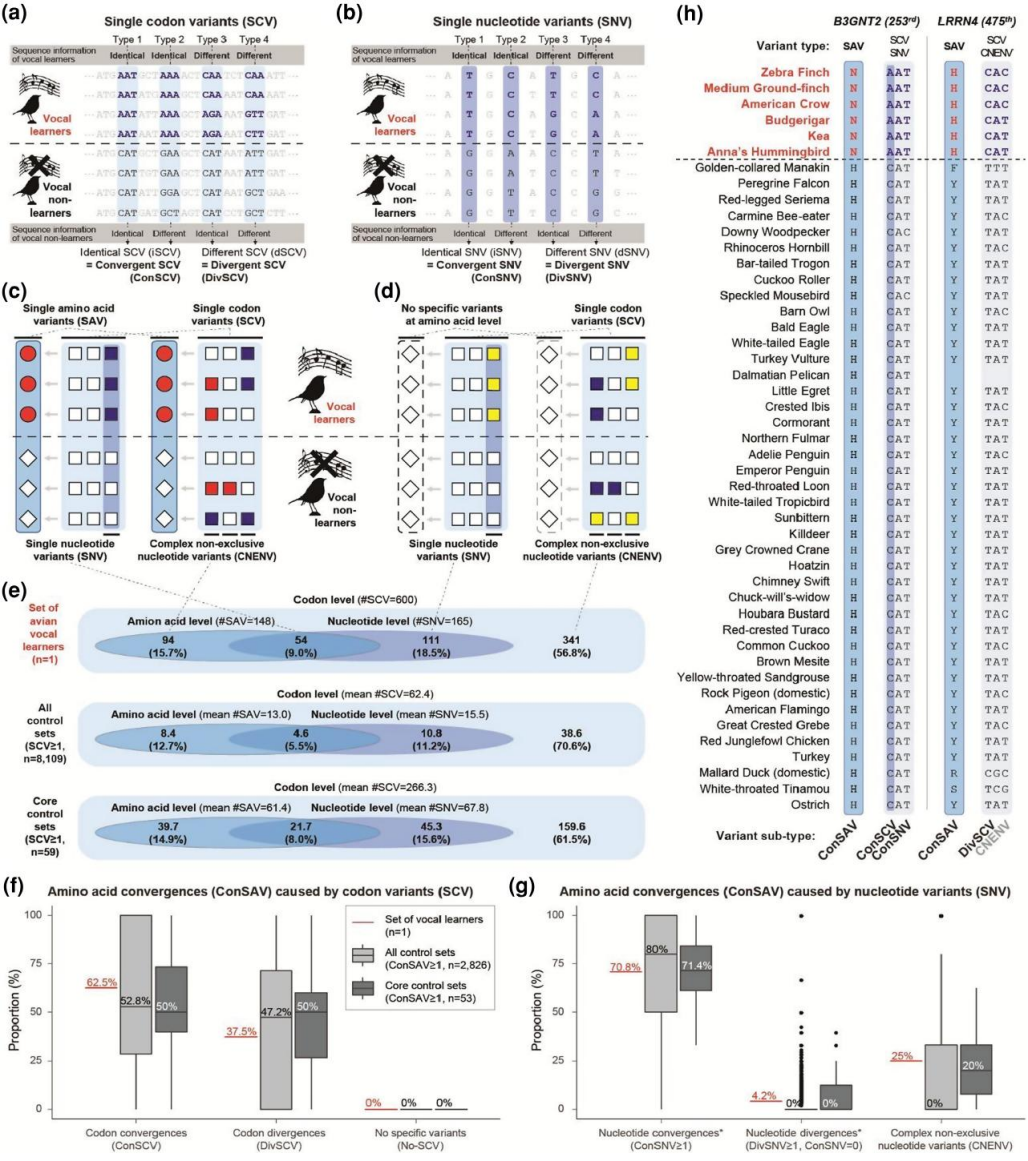
Codon and nucleotide sources of amino acid convergences. a) Example illustration of four types of SCVs. b) Example illustration of four types of SNVs. c) Concept of SAVs explained by nonsynonymous SCVs and SNVs. Left case, ConSAVs caused by nonsynonymous ConSCVs with ConSNVs at a homologous nucleotide site. Right case, ConSAVs caused by nonsynonymous DivSCVs with CNENVs at different sites in the codon of different species. d) Concept of nonexclusive amino acid variants explained by synonymous substitutions between species, which do not cause amino acid changes. Left case, same amino acids caused by synonymous ConSCVs with ConSNVs at a homologous nucleotide site. Right case, same amino acids caused by synonymous DivSCVs with CNENVs. e) Venn diagrams of the different subsets of nonsynonymous and synonymous SCVs and SNVs outlined in c) and d), in avian vocal learners (*n* = 1), control sets with at least one SCVs (*n* = 8,109), and the core control sets (*n* = 59). f and g) Boxplots of proportions (median and range) of types of codon and nucleotide sources causing amino acid convergences. h) Examples of amino acid convergences among vocal learners (ConSAVs) originating from different types of nucleotide variants at the same site (in *B3GNT2*) or different sites (in *LRRN4*). Red bold text, avian vocal learners. Sky blue boxes, sites with SAVs; light sky-blue boxes, SCVs; dark sky-blue box, SNVs.

To trace the codon and nucleotide sources of the convergent amino acid substitutions, we performed ConVarFinder in codon and nucleotide modes on avian codon alignments of the same 8,295 protein-coding genes and species combinations, to identify convergent and divergent single codon variants (ConSCVs and DivSCVs, respectively, Fig. 3a) and convergent and divergent single nucleotide variants (ConSNVs and DivSNVs, respectively, Fig. 3b). Theoretically, nonsynonymous nucleotide substitutions that cause convergent amino acid substitutions can be the result of simple single nucleotide variants (SNVs) or multiple complex nonexclusive nucleotide variants (CNENVs; Fig. 3c). Synonymous variants on the other hand do not lead to convergent or DivSAVs (Fig. 3d).

In the 4,519,041 homologous codons from 13,557,123 homologous nucleotides among the 8,295 orthologous genes in birds, we found 600 vocal learner–specific single codon variants (SCVs) (Fig. 3e). Of these 600, all 148 nonsynonymous vocal learner–specific SCVs found explained all 148 vocal learner–specific SAVs (Table S1). Of these 148 nonsynonymous vocal learner–specific SCVs, 54 (36%) resulted from SNVs and 94 (64%) resulted from CNENVs at different codon positions (Fig. 3e). By comparison, of the remaining 452 synonymous (out of 600) vocal learner–specific SCVs, 111 (24%) resulted from SNVs and 341 (76%) resulted from CNENVs (Fig. 3e). Among the 8,238 control species combinations, the mean number of convergent SCVs differed from that seen in vocal learners, but the relative proportions (%) of the types of SCVs were similar (Fig. 3e).

For the 24 amino acid sites with identical ConSAVs in vocal learners, 15 (62.5%) were caused by ConSCVs and 9 (37.5%) were caused by DivSCVs (Fig. 3f). Their nucleotide sources were more complex, where of the 24, 17 (70.8%) were caused by ConSNVs, 1 (4.2%) by DivSNVs, and 6 (25%) by CNENVs (Fig. 3g). Again, the proportional values of the codon and nucleotide sources for the identical convergent amino acid substitutions in vocal learners fell within the ranges we found for the control species combinations (Fig. 3e to g).

An example of a simple case with nucleotide convergence causing identical amino acid convergence in vocal learners is the ConSAV site of *B3GNT2* mentioned earlier, where all vocal learners have the same convergent nucleotide (A) in the first position of the codon AAT for asparagine (N) and all vocal nonlearners have the CAT or CAC for histidine (H; Fig. 3h). An example of a complex case with nucleotide variants at different sites causing identical amino acid convergence is the ConSAV site of *LRRN4*, where all vocal learners have the CAC or CAT codon for histidine (H), while nearly all vocal nonlearners have either the TAT or TAC codon for tyrosine (Y) and two have more complex changes of TCG and GCG codons (Fig. 3h).

These findings suggest that the convergent amino acid evolution can originate from either convergent nucleotide variants or divergent and complex nucleotide variants at the same or different positions within the codon.

### Codon and Nucleotide Variants Are Also Correlated With the Product of MRCA Branch Lengths

**Fig. 4.**
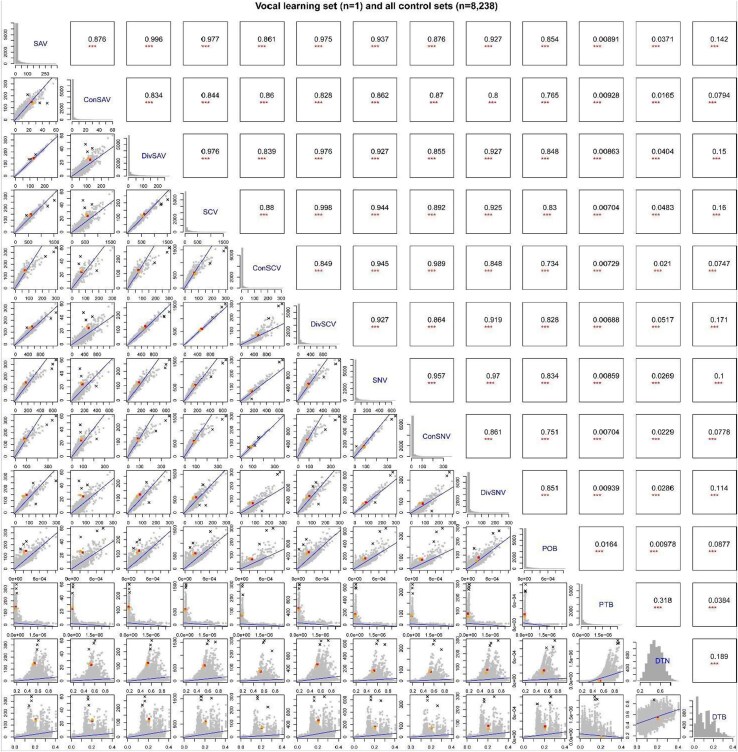
Correlation analyses among various convergent evolution, molecular, and phylogenetic parameters. Histograms on the diagonal of the matrix are of frequencies of each convergent variant and values of each phylogenetic feature visualized, and list of all the names of the variables compared. Graphs along the lower left of the matrix are regression plots; black, gray, orange, and red spots indicate all control sets (*n* = 8,237), the closest control set of vocal learners (*n* = 1), and the set of avian vocal learners (*n* = 1), respectively. The statistical values for these regression graphs are in upper diagonal matrix, *P*-values and adjusted *R*^2^ (*P < 0.05, **P < 0.01, and ***P < 0.001). Black lines and black “X” marks indicate regression lines and outliers, respectively. Correlations of core control sets are shown in Fig. S4. Color coding is the same as in Fig. 1d.

To assess whether phylogenetic or other factors influence convergent codon and nucleotide variant numbers in different polyphyletic species combinations as they did for amino acids, we performed comparative correlation tests between ten variables: three evolutionary types (convergent, divergent, and all); three molecular types (amino acid, codon, and nucleotide); and four phylogenetic features (POB, PTB, DTB, and DTN). As expected, one or more of these variables were correlated with each other, in all control (Fig. 4) and core control species combinations (Fig. S3). However, for the phylogenetic variables, the POB showed the strongest correlation with convergent and divergent evolutionary types and at all three molecular levels, whereas the other phylogenetic variables (DTB, DTN, and PTB) had either weaker correlations or were not correlated at all (Figs. 4 and  S3). Correlations were overall stronger for the full control species combinations (Figs. 4 and  S3), presumably due to the higher number of species combinations than in the core control set. Like amino acid convergences (Fig. 2), the number of convergent vocal learner–specific codon or SNVs still were not among the outliers in any of the correlations assessed (Fig. 4).

### Fixation and Positive Selection of Convergent Amino Acid Sites in Vocal Learning Birds

Like most studies, we have one genome per species, and thus some of the variants identified could instead be due to population differences within a species as opposed to species differences. To check for possible population differences, we scanned the dbSNP database of zebra finch (*n* = 1,257 samples; build 139) a vocal learner and chicken (*n* = 9,586 samples, build 145) a vocal nonlearner. At the 148 SAV sites found in vocal learners, zebra finches showed complete fixation without any nonsynonymous polymorphisms. Chicken only showed one missense single nucleotide polymorphism (SNP) in the *OTOA* gene (c.2581A>G, p.Thr861Ala), resulting in an amino acid change identical to that of vocal learners (Fig. S4). We also validated fixation of the convergent substitutions in *DRD1B* by polymerase chain reaction (PCR) of genomic DNA and sequencing from three male and three female zebra finches and three male and three female chickens (Fig. S5; *n* = 6 total of each species). These findings indicate that the vast majority (99.3% thus far) of the SAVs we identified in vocal learners are the result of true species-specific variants that are fixed and thus presumably selected upon.

To check for positive selection, we performed dN/dS analyses with the branch-site model on the 24 ConSAV sites of these genes in the vocal learning species. We found that 10 of the 24 sites (∼42%) showed signs of positive selection (likelihood ratio value [*D*] >0, posterior probability >0.5, dN/dS *ω*_2_ values of ancestral branches of each vocal learning clade >1). With a stricter statistical significance (adjusted *P* < 0.05), only 3 of 24 (12.5%) were under positive selection. We compared the proportions of amino acid convergences in the closest control set (songbirds, parrots, and swifts) under positive selection, and found similar proportions of 12 out of 26 (46%; Swift-ConSAV genes) under positive selection with the cutoff for the likelihood ratio test (LRT, *D*-value) >0 and 6 genes (23%) with the stricter significance (adjusted *P* < 0.05). These findings suggest that a subset of sites with amino acid convergences in vocal learners have been positively selected, but like the number of convergent sites, this positive selection rate may be the background rate on convergent substitutions as seen in the closely related control species combination without a known convergent trait.

### Post Hoc Analyses Reveal Rifleman Variants Are Shared More With Vocal Nonlearners

Although rifleman, a presumed vocal nonlearner, was excluded from the initial ConVarFinder analyses, by doing so we could ask in an unbiased way whether its sequences are more similar to vocal nonlearners or vocal learners. We applied principal component analysis (PCA) and phylogenetic analysis for the 148 vocal learner–specific SAV sites and the subset of 24 ConSAV sites unique to vocal learning clades. Despite being more closely related to songbirds, the pattern of these 148 sites in rifleman clustered the species among the vocal nonlearners in both the PCA and consensus tree (Fig. S6a and c). For the 24 ConSAV subset, the pattern in rifleman was separate from the two groups but still closer to vocal nonlearners in the PCA (Fig. S6b), while it clustered as an outgroup sister taxon of vocal learning birds instead deeper as a sister branch to songbirds within the consensus tree (Fig. S6d). These results support the assumption that rifleman is a vocal nonlearner and further suggest that despite the number of variants and their positive selection not differing from expectation, the actual substitutions and their associated genes could be related to the convergent trait of vocal learning.

### Biological Functions of Genes With Amino Acid Convergences

**Fig. 5.**
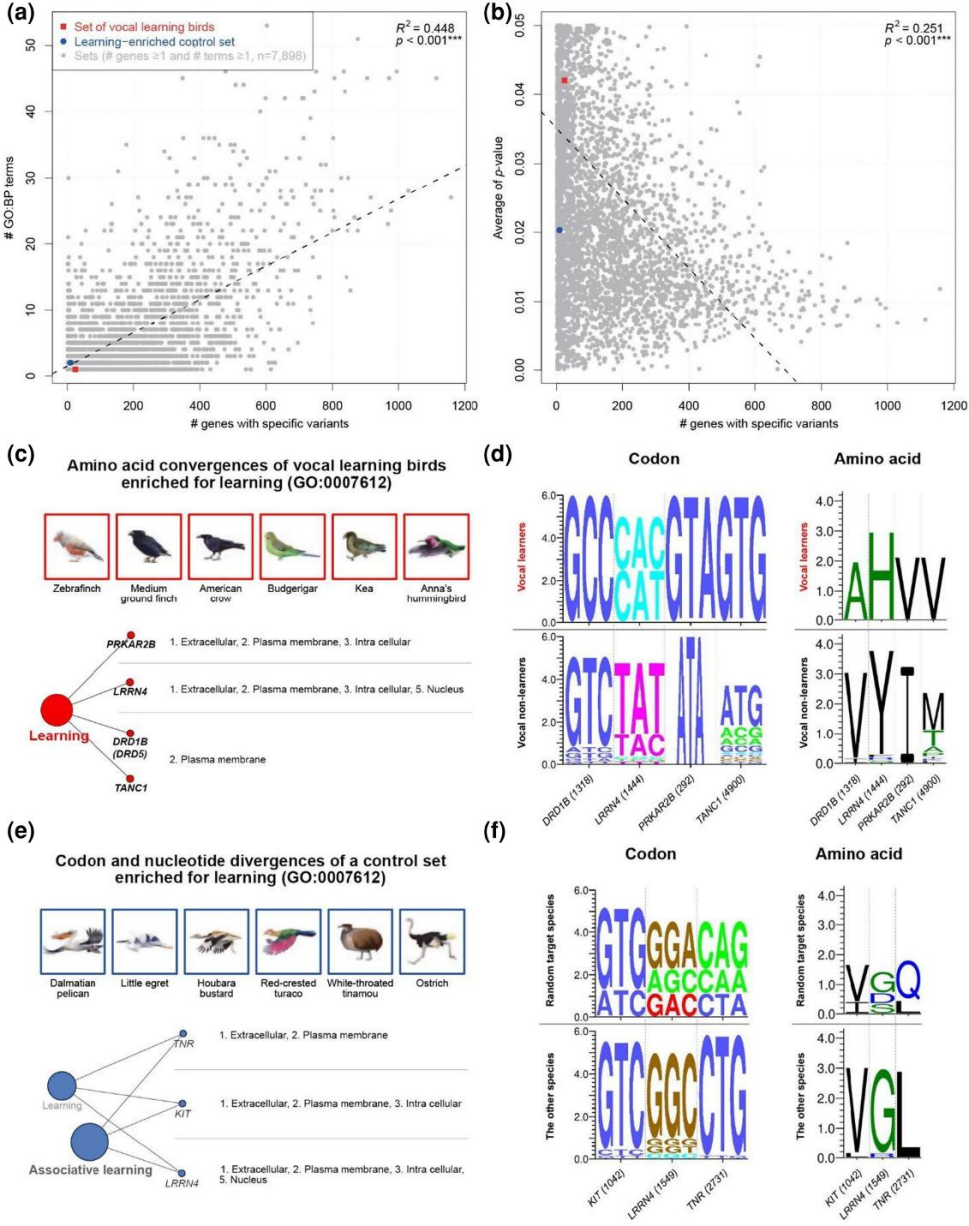
Functional ontology of genes with amino acid convergences. a) Correlation plot between the number of significantly enriched GO terms and the number of genes with one or more convergent variants in each species data set with nine convergent sequence types (SAVs, ConSAVs, DivSAVs, SCVs, ConSCVs, DivSCVs, ConSNVs + DivSNVs, ConSNVs, and DivSNVs) (*n* = 53,058 returned GO terms). b) Correlation plot between averages of *P*-values and number of genes of the same data sets. c) GO and network results for four learning-associated genes with amino acid convergences (ConSAVs) in vocal learners (adj*. P* < 0.05). d) Codon and amino acid sequence logos of vocal learner–specific ConSAV sites in genes associated with learning. e) GO and network analysis for learning-associated genes with codon and nucleotide divergences (DivSCVs and DivSNVs) of a control species combination (adj*. P* < 0.05). f) Codon and amino acid sequence logos of DivSCV and DivSNV sites from a control species set in genes associated with learning.

To investigate the biological functions of genes with convergent sequence variants in vocal learners and in other species combinations, we performed gene ontology (GO) analyses for 53,058 gene lists with one or more of each type of sequence variant for vocal learners and all 8,238 control species combinations. Among them, at least one significant (adjusted *P* < 0.05) GO term was found for 7,901 gene lists (14.9%). For these 7,901 lists, we found a positive correlation between the number of significant GO terms and the number of genes with convergent variants (Fig. 5a), while a weaker negative correlation between the average adjusted *P*-value of significant GO terms and the number of genes with convergent variants (Fig. 5b). This result means that the lower the number of convergent genes, the less likely of finding a significant shared GO function. The vocal learners were at the lower end of this correlation, and though they did not have significant GO enrichment for their total 148 full SAV gene list, they did so for their fewer 24 ConSAV gene list, which was significantly enriched for “learning” (GO:0007612, adjusted *P* = 0.042). Four genes were responsible for this enrichment: *DRD1B*, *LRRN4*, *PRKAR2B*, and *TANC1* (Fig. 5c). *PRKAR2B* and *DRD1B* modulate the cAMP signaling pathway for learning (including vocal learning) via cAMP response element binding protein (*CREB1*), whereas *LRRN4* and *TANC1* influence memory through other molecular pathways (Fig. S7) (Sunahara et al. 1991; Solberg et al. 1992; Bando et al. 2005; Han et al. 2010; da Silva et al. 2012; Kandel 2012; Wong et al. 2012; Abe et al. 2015; Rangel-Barajas et al. 2015). The amino acid convergences of *DRD1B*, *PRKAR2B*, and *TANC1* were caused by codon convergences (ConSCVs) in vocal learners, while that of *LRRN4* were caused by codon divergences (DivSCVs) with complex nucleotide variants (CNENVs; Figs. 3h and 5d).

Out of the 8,238 control species combinations, only one had 2 gene lists, DivSCVs and DivSNVs, that showed significant enrichment for “learning” (GO:0007612, both adjusted *P*-values = 0.02); the associated set of species (Fig. 5e) did not include any vocal learners, but another convergent variant in *LRRN4* contributed to this functional enrichment (Fig. 5f). The findings indicate that a subset of genes with identical convergent variants in vocal learners is enriched for learning functions, and this convergent enrichment is rare.

### Candidate Genes Supported by Meta-Analyses for Vocal Learning

We next tested if there was any relationship between genes with amino acid convergences specific to vocal learning birds and previous candidate genes implicated in vocal learning behavior, and/or vocal learning brain regions. Out of 8,295 singleton orthologous genes, we analyzed 6,932 genes with same gene symbols in at least one of six gene sets from 3 types of data sets:(i) 786 genes that are targets of the *FOXP2* transcription factor (Lachmann et al. 2010; Lovell et al. 2020); (ii) 1,769 genes (25.5%) that are up- or downregulated in song learning nuclei of the zebra finch in response to singing (Hilliard et al. 2012a; Hilliard et al. 2012b; Whitney et al. 2014; Lovell et al. 2020); and (iii) 3,001 differentially expressed genes (DEGs, 43.3%) that have specialized up- or downregulated expression in zebra finch song nuclei or human speech regions compared with their surrounding nonvocal motor brain regions (Lovell et al. 2018; Gedman et al. 2022). Of the total 6,932 genes, 4,353 were present in at least one gene data set (Table S2). Using a hypergeometric test, we calculated whether there were any overlapping enrichments between these gene sets and one control combination that included a swift switched with the hummingbird.

**Fig. 6.**
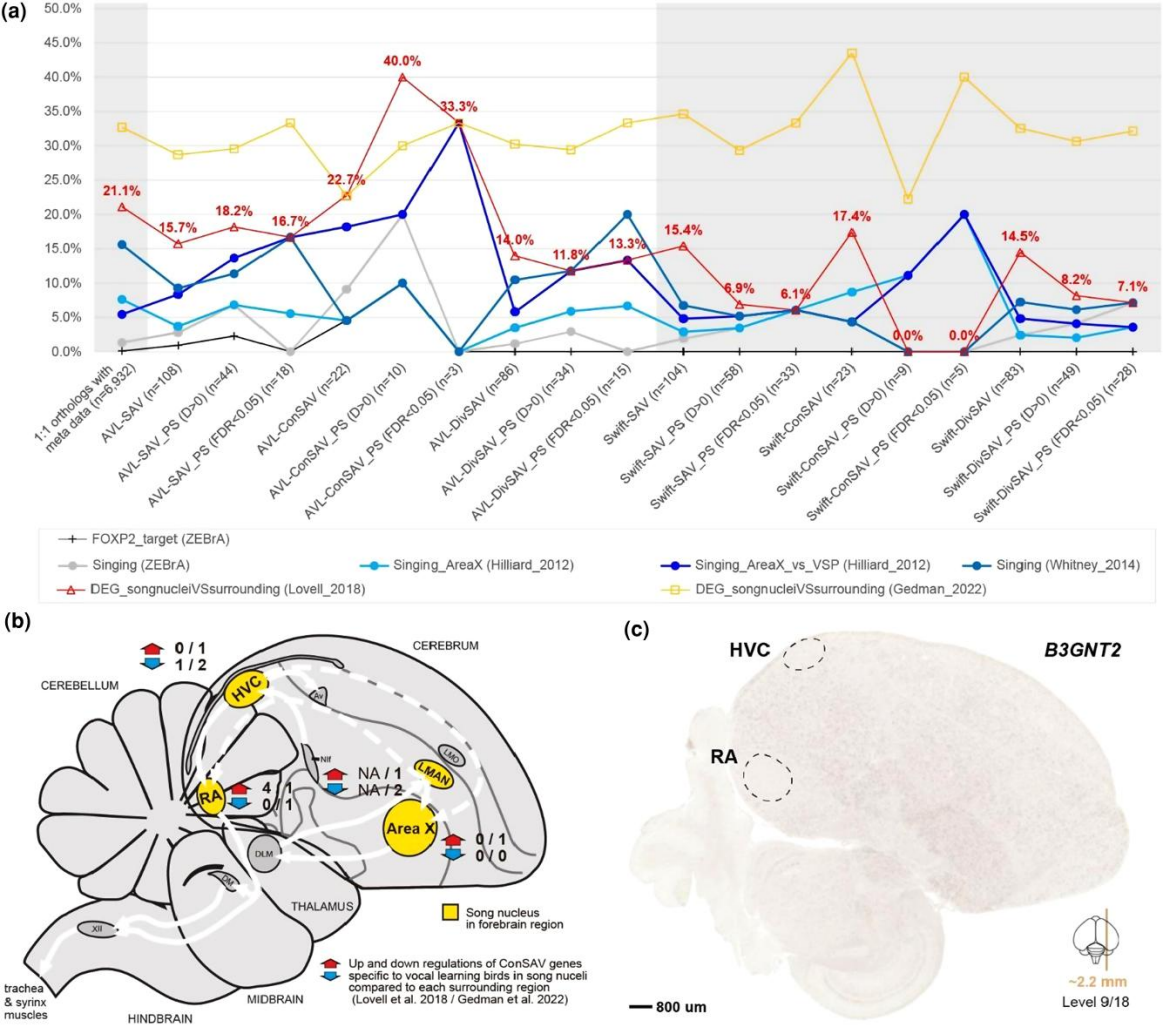
Meta-analysis for overlapping genes sets in vocal learning species. a) Proportions of candidate genes (%) with convergent amino acid changes (SAVs, ConSAVs, and DivSAVs) in vocal learning species (white background) or control sets of species (gray background) that overlap with gene sets implicated in vocal learning behavior and brain circuits (color lines). The latter data sets include three major types: targets of the *FOXP2* gene; singing-regulated genes in song nuclei of zebra finch; and differentially expressed genes in song nuclei compared with their surrounding nonvocal regions of zebra finch, all collected from published sources mentioned in fig. S8. For the convergent gene sets, we also break them down into three types of SAVs under positive selection with different stringent statistical levels: no filtering for positive selection, likelihood of positive selection (*D* > 1), and likelihood of positive selection with an FDR-adjusted *P-*value <0.05. b) Songbird brain diagram showing the song learning system. Yellow, forebrain song learning brain regions that had their gene expression profiled; gray, other song learning nuclei; white arrows, connections between the song nuclei; red-up arrow and blue-down arrow, numbers of the subset of ConSAV genes, supported by two independent data sources (Lovell et al. 2018; Gedman et al. 2022). c) *B3GNT2* mRNA expression patterns in zebra finch HVC and RA. This in situ hybridization image was retrieved from the ZEBrA database (Lovell et al. 2020).

Out of 306 comparisons, 9 showed significant enrichments with 5 gene sets before multiple testing corrections (*P*-value <0.05, Fig. S8); none showed statistical significance after false discovery rate (FDR) correction (adjusted *P*-value = 1). Of these 9 enrichments, 8 were with the full 148 or 24 vocal learner ConSAV genes with identical amino acids in vocal learners, none with the DivSAV of vocal learners, and only 1 for the control group of switching swift with the hummingbird (Fig. S8). The strongest hypergeometric correlation (*P* = 0.01) was with the 10 positively selected out of the 24 ConSAV genes, which overlapped with *FOXP2* transcription factor targets and the song nuclei singing-regulated genes collated in the ZEBrA database. The subset of six genes that survived FDR correction for positive selection were also enriched for genes differentially expressed in brainstem vocal motor neurons (nXIIts) versus SSP (supraspinal nucleus) neck motor neurons (Pfenning et al. 2014; Lovell et al. 2018); nXIIts is used to produce both innate and learned vocalizations by directly controlling the syrinx (Simpson and Vicario 1990; Maat et al. 2014). We also calculated the proportions (%) of genes with variants in the vocal learner and control sets that overlap with these gene data sets, and found that the 24 ConSAV gene set of vocal learners had the highest proportional overlap with singing-regulated genes in song nuclei and with genes that have specialized expression in song nuclei (Fig. 6a). A summary of their singing-regulated and specialized expression patterns in song nuclei is shown in Fig. 6b and Table 1. Again, out of the 24 vocal learner ConSAV gene set, 10 had at least one relationship to vocal learner associated gene expression data sets (Fig. 6b), including the 6 with higher likelihood for positive selection in vocal learners: *B3GNT2, DRD1B, FNDC1, PIK3R4, PRKAR2B, and SMPD3* (Table 1). The top candidate gene supported by all three major types of data sets, *B3GNT2*, has specialized downregulation in HVC and RA of adult zebra finches (Lovell et al. 2020) (Fig. 6c). Two of these six genes, *DRD1B* and *PRKAR2B*, also contributed to the GO finding for learning functions (Fig. 5c and d). Further, *DRD1B* has specialized upregulation in adult Area X compared with the surrounding striatum (Kubikova et al. 2010) (Fig. 6d).

**Table 1:** Candidate genes with convergent amino acid changes under positive selection in vocal learning clades and supported by multiple lines of evidence for vocal learning

		AA profiles		Positive selection			FOXP2_targets	Singing				DEG_song nuclei_vs_surround	
Symbol	Pos_AA	Avian vocal learners	Avian vocal nonlearners	dN/dS (*ω*) FG	Likelihood ratio (*D*)	Adj. *P* (FDR)		AreaX (ZEBrA)	AreaX (Hilliard et al. 2012a)	AreaX_vs_VSP (Hilliard et al. 2012b)	All songnuclei (Whitney et al. 2014)	AX, HVC, RA (Lovell et al. 2018)	All song nuclei (Gedman et al. 2022)
*B3GNT2*	253	N	H	3.3	1.1	2.8.E−01	+	+	+	…	AXdown	HVCdown	HVCdown /RAdown
*DRD1B (DRD1B)*	416	A	I,V	3.7	1.2	2.7.E−01	…	+	…	…	…	RAup	LMANdown
*FNDC1*	1034	S	G	6.7	4.3	6.4.E−02	…	…	…	+	…	RAup	…
*PIK3R4*	671	C	R	10.4	4.9	4.9.E−02	…	…	…	…	…	RAup	…
*PRKAR2B*	32	V	I,-	295.8	25.4	3.6.E−06	…	…	…	+	…	…	…
*SMPD3*	307	C	Y,-	14.3	6.0	3.4.E−02	…	…	…	…	…	…	AXup

“Pos_AA” column shows amino acid position in gene-wide multiple sequence alignments. “AA avian vocal learners” and “AA avian vocal nonlearners” columns show amino acid profiles of vocal learning and vocal nonlearning groups, respectively. “dN/dS (*ω*) FG” column shows positive selection values (*ω*>1) of ancestral branches of vocal learning clades relative to foreground branches. “Likelihood ratio (*D*)” column shows likelihood ratios between null and alternative hypothesis assuming neutral and negative selection (*D* ≤ 0) and positive selection (*D* > 0). “Adj. *P* (FDR)” column shows adjusted *P*-value assuming positive selection (*ω*>1) applied with FDR correction (adj. *P* < 0.05). The other columns show zebra finch gene data sets for three major types of gene candidates for vocal learning: target genes of the *FOXP2* gene, in the ZEBrA database (Lovell et al. 2020); singing-regulated genes in Area X from the ZEBrA database (Lovell et al. 2020); singing regulated in Area X from Hilliard et al. (2012a); singing regulated in AreaX versus VSP from Hilliard et al. (2012b); singing regulated in all four major song nuclei in a 7-h time course from Pfenning et al. (2014) and Whitney et al. (2014); differentially expressed genes in four song nuclei compared with their surrounding regions using microarrays collated by Lovell et al. (2018); and differentially expressed genes in four song nuclei compared with their surrounding regions using RNA-seq by Gedman et al.(2022).

### Most Convergent Substitutions in Vocal Learners Remain With Inclusion of More Species

Until now, we investigated convergent amino acid sequences among 48 species from the first phase of the B10K project. While we were completing this study, the consortium additionally released Illumina short-read–based genome assemblies and constructed genome-wide alignments of a total of 363 avian species representing 218 out of 236 bird families, providing an opportunity to check whether the convergent patterns still exist in the more densely sampled data set of the avian family tree (Feng et al. 2020; Stiller et al. 2024). As revealed in a study on marine mammals (Thomas et al. 2017), the number of convergent mutations in a polyphyletic clade is expected to decrease as more species are included.

**Fig. 7.**
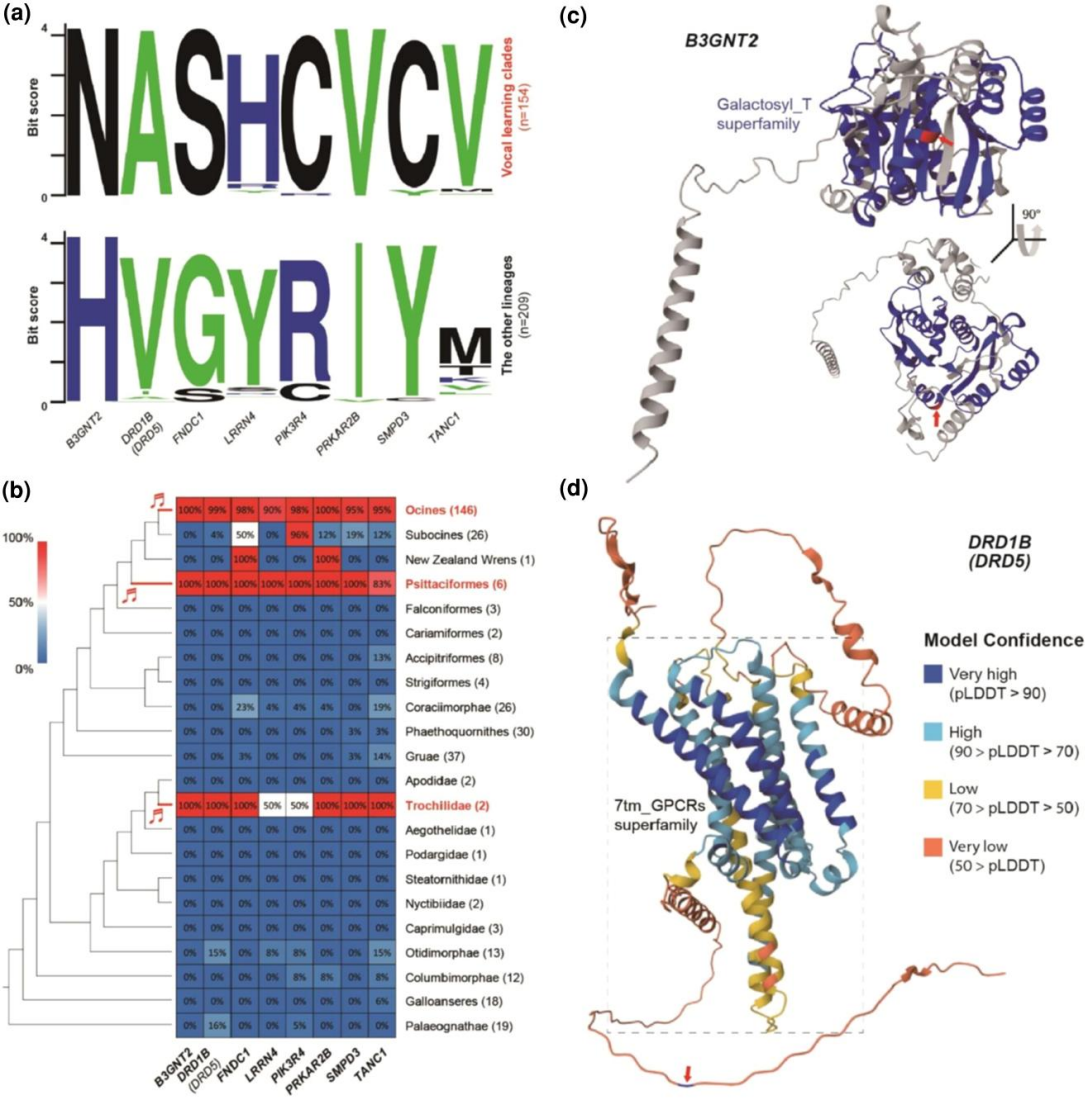
Enrichments of vocal learner type amino acid patterns in 363 bird species. a) Sequence logos of amino acid profiles convergent sites in 8 genes comparing 154 vocal learning species and 209 species considered vocal nonlearners. Color codes applied for positively charged residues (K, R, H) as blue, negatively charged residues (D, E) as red, hydrophobic residues (A, F, G, I, L, P, V, W, Y) as green, and the other residues as black. b) Heatmap of vocal learner–type amino acid enrichments for 363 bird species grouped into 22 avian ordinal or higher classification lineages. Numbers in parentheses of each lineage indicate the numbers of species. The color scale shows the proportions of the vocal learner–type amino acid patterns assigned from the representative vocal learner, zebra finch, in each lineage. On the left cladogram, red bold branches indicate the most recent ancestral branches of vocal learning clades. c) Estimated protein structure of the *B3GNT2* gene in zebra finch. The red arrow points to the ConSAV vocal learner site, which is located within the galactosyl-transferase (galactosyl-T) superfamily domain (navy blue). d) Estimated protein structure of the *DRD1B* gene in zebra finch. The red arrow points to the ConSAV vocal learner site located on an intracellular domain. The dashed box highlights the seven-transmembrane G protein–coupled receptors (7tm_GPCR). The color legend indicates the level of confidence of the protein substructure. Both structures were estimated and visualized using AlphaFold2 (Jumper et al. 2021).

We confirmed that known outlier vocal learning birds (*Procnias nudicollis* [bare-throated bellbird] a suboscine (Kroodsma et al. 2013) and *Biziura lobata* [musk duck] [Ten Cate and Fullagar 2021]) not within songbirds, parrots, or hummingbirds, were absent in 215 potential vocal nonlearning families. There were 154 species from the three known vocal learning orders: songbirds, parrots, and hummingbirds. We analyzed 8 key candidate sites with vocal learner ConSAVs supported by multiple lines of evidence (Table S3). Although exclusivity to known vocal learners was reduced, the convergent amino acid patterns were still highly enriched in the vocal learners: 89.6% to 100% of the 154 vocal learning species still had the substitution and 86.1% to 100% of the 209 species regarded as vocal nonlearners did not have it (Fig. 7a and b; Table S4). For example, *B3GNT* still had mutually exclusive variants convergently unique to 154 species from the 3 vocal learning clades compared with the other 209 species (Fig. 7a and b). For *DRD1B*, 153 of the 154 vocal learning birds maintained the alanine (A) substitutions; the exception was the noisy scrubbird (*Atrichornis clamosus*) in Australia, once thought to be extinct, which lost the pattern to valine (V); only 1 to 2 species in 3 bird lineages (a suboscine, Otidimorphae, and Palaeognathe) of 209 vocal nonlearners had the substitution seen in vocal learners. For *LRRN4*, one of the two hummingbird species lost the substitution seen in all the remaining 153 vocal learners (Fig. 7b). For *TANC1* an incomplete genome sequence caused a genome alignment indel error for the two hummingbirds, but on manual inspection both species had the vocal learner substitution. These findings indicate that although exclusive substitutions in vocal learners are rare, extremely conserved substitutions are present.

### Evaluating the Biochemical Impact of Amino Acid Changes Specific to Avian Vocal Learners

To assess the functional locations of amino acid modifications unique to avian vocal learners in protein structure, we explored functional protein domains containing vocal learner ConSAVs (Table S5). Particularly for two key candidate genes, *B3GNT2* and *DRD1B*, we analyzed protein structures using AlphaFold and the biochemical properties of the convergent residues (see Fig. 7c and d). The convergent residue in *B3GNT2* was in the galactosyl-transferase superfamily domain, which catalyzes posttranslational modifications in the Golgi apparatus. The substitution can be classified as a radical amino acid replacement, transitioning from a basic histidine (H) with a positively charged side chain in vocal nonlearners to an acidic asparagine (N) with a polar uncharged side chain in vocal learners. The convergent residue in *DRD1B* was located in the intracellular domain, outside the seven-transmembrane G protein–coupled receptor (7tm_GPCR) domain. This amino acid change in *DRD1B* was more conservative, from valine (V) to alanine (A), due to their similar aliphatic properties and hydrophobic side chains. These two examples indicate that changes in the convergent amino acids in vocal learners could have functional consequences.

## Discussion

Amino acid substitutions contribute to changes in protein functions, and thereby evolution of various traits including human spoken language (Lai et al. 2001; Enard 2011; Berwick et al. 2013). To assess properties of possible convergent amino acid substitutions among species that have a component of spoken language, the rare trait of vocal learning (Jarvis 2019), we developed new tools to scan genomes and amino acid alignments for convergent variants in different polyphyletic species combinations. In doing so, we not only discovered convergent variants in vocal learners in genes enriched for learning functions, but a principle of convergence, being positive correlations between the frequencies of molecular convergences at the amino acid, codon, and nucleotide levels with the product of the MRCA (origin) branch (POB) lengths. This suggests that certain aspects of convergence are predictable. Vocal learners did not have a higher preponderance of convergent variants above background levels. To explain our findings, we propose a hypothesis of selection for convergent traits on a background of convergent substitutions.

When searching for convergent substitutions among species, we believe our multiwise species comparison approach and the POB is more informative than past approaches. Previous studies found correlations between convergent and divergent variants (called convergent and divergent substitutions in those studies) between pairs of species (Castoe et al. 2009; Thomas and Hahn 2015). We find that such a relationship exists also at higher dimensional species combinations. Castoe et al. 2009 and Foote et al. 2015 called such a relationship “neutral convergence” and Thomas and Hahn 2015 called it “background convergence,” which we also do. We define this background convergence as convergence by chance over time. Several other studies found that the rate or number of convergent substitutions decreases with increasing branch distance between two polyphyletic species (Goldstein et al. 2015; Zou and Zhang 2015). But the correlations were not strong, and here, we also fail to find strong relationships of genetic convergence with such phylogenetic parameters including summations of branch lengths. Rather, we find that the POB has a much stronger correlation with the numbers of convergent variants in polyphyletic clades. This result suggests that the deeper in time their common ancestor, the more likely the evolution of detectable convergences at the amino acid, codon, and nucleotide levels.

Against this phylogenetic background of convergence, our positive selection, gene function, and gene expression associations suggest that selection occurs on some of these convergent substitutions to contribute to evolving novel, convergent traits, in our case vocal learning. According to this hypothesis, it is not about how many genes show convergence, but which specific genes and specific nucleotide sites show convergence may be the more important factors to consider.

The learning-related functions of the genes with ConSAVs in vocal learners include the following: *DRD1B* that regulates striatal activity associated with learning (da Silva et al. 2012; Wong et al. 2012), *LRRN4* that affects long lasting memory (Bando et al. 2005); *TANC1* that regulates dendritic spines and spatial memory (Han et al. 2010), and protein kinase cAMP-dependent type ii regulatory subunit beta (*PRKAR2B*), an enzyme that activates cAMP-dependent protein kinase (PKA) inside the cell (Solberg et al. 1992). Blocking of PKA inhibits learning, including vocal learning (Abe et al. 2015), namely through the *CREB1*, which regulates genes that convert short-term memories into long-term memories (Kandel 2012). *DRD1B*, through its G protein, also regulates activity of adenylyl cyclase's synthesis of cAMP in the cell membrane (Sunahara et al. 1991; Rangel-Barajas et al. 2015).

The fact that the broader inclusion from 48 to 363 (7.5-fold) species resulted in 0 to 4 vocal nonlearner species containing 8 mutually exclusive substitutions initially found only in vocal learners, indicates that these substitutions are still highly conserved in vocal learners. Should future mechanistic studies prove that these substitutions contribute to the vocal learning trait, it would then be consistent with vocal learning not being an exclusive dichotomous trait, but a continuous trait, where some so-called vocal nonlearners have rudimentary aspects of the trait with a subset of the substitutions. Experiments in mice, suboscine birds, and other so-called vocal nonlearning species (Arriaga et al. 2012; Petkov and Jarvis 2012; Liu et al. 2013; Jarvis 2019; Fischer et al. 2020; Ten Cate and Fullagar 2021) have found various degrees of rudimentary features in behavior, neural connectivity, and gene expression seen in vocal learners. Even within well-established vocal learning clades, there are differences in vocal learning abilities, with some species able to imitate a few hundred words of human speech and other species learn just one short song of its own species for its entire life (Kroodsma 1983). A vocal learning continuum across species within the songbird lineage has recently been shown to correlate positively with problem-solving skills (Audet et al. 2023). To test whether our findings in the more expansive set of 363 species represent a partial continuum or additional advanced vocal learning species will require more behavioral and neural investigations into the vocal behavior of such species. Another alternative is that the combination of these convergent substitutions occurring together in a species is more important than the presence of any one of the substitutions alone. Our findings provide candidates for testing either of these nonmutually exclusive hypotheses.

From exclusive substitutions between vocal learners and nonlearners, *B3GNT2* emerges as the one with the most promising candidate variant. The specific radical change from basic histidine to acidic asparagine in *B3GNT2* occurs within the catalytic domain for posttranslational glycosylation. Given *B3GNT2*'s pivotal roles in brain development and axon guidance related to adenylate cyclase 3 (Knott et al. 2012; Xie et al. 2024), combining an effect of this convergent radical substitution with the decreased gene expressions in the HVC and RA regions of the adult zebra finch brain might influence vocal learning ability.

Often coding and regulatory genomic sources of trait evolution are pitted against each other as alternatives (Carroll 2005), but our findings suggest that they could synergistically influence evolution of each other. Preliminary studies in our group find the promoter regulatory region of *DRD1B* has differential open chromatin activity within Area X versus the surrounding striatum, and the striatum further has differential open chromatin differences with the other cortical-pallial regions and song nuclei (Rhie et al. 2021; Kim et al. 2022). These GC-rich regulatory regions are better sequenced and assembled in long-read–based genomes, like those that are currently under progress for all vertebrate orders by the Vertebrate Genomes Project (Rhie et al. 2021). The assemblies we used here are from the 48 avian genomes generated from the first phase of the Avian Phylogenomics Project (Jarvis et al. 2014; Zhang et al. 2014), and the 363 species from the second phase (Feng et al. 2020), which are mostly short-read-based and missing many of the GC-rich regulatory promoter regions (Rhie et al. 2021; Kim et al. 2022). The new long-read assemblies, however, do not yet have all bird or vertebrate orders represented. When they are completed, we will be able to further analyze possible convergent relationships between coding and noncoding regulatory regions.

In future studies it will be useful to test whether similar or different principles apply for nucleotide convergence and the POB in noncoding regions that regulate differential gene expression. It will also be useful to determine whether the convergent principles we identified here are specific to birds or are more widespread across life forms. Vocal learning species also share other convergent traits besides vocal learning (Naguib and Riebel 2014; Nowicki and Searcy 2014; Mason et al. 2017; Jarvis 2019), and the identified genes and their functions in the surrounding brain regions could be associated with these other traits. Overall, our study generates new hypotheses for functions of genetic changes enriched in vocal learners and for principles of convergence.

## Materials and Methods

### Multiple Sequence Alignments of Singleton Orthologous Genes in Birds

In our preliminary studies, the Avian Phylogenomics Project (now the Bird10K project) defined 8,295 singleton orthologous gene sets across 48 avian species, and constructed a total genomic evidence phylogenetic avian family tree consisting of at least 34 orders (Jarvis et al. 2014, 2015; Zhang et al. 2014). This 1:1 orthologous gene set was identified by reciprocal best blast hits and synteny, using two species as a reference: chicken and zebra finch. They were then aligned across all species using SATé + MAFFT and SATé + Prank, for both nucleotide and amino acid sequences. Alignment frameshift errors were corrected when translating into amino acid sequence alignments. This resulted in detection of 4,519,041 amino acid and 13,557,123 nucleotide homologous sites. In our previous analyses for amino acid substitutions, we used gBlocks (Castresana 2000) to remove poorly scored alignments with sequence divergences and columns with gaps in at least one species included. However, here we found that this was too aggressive, removing 65% of the aligned sequences. For example, vocal learner–specific amino acid substitutions of *DRD1B* were excluded because of gaps in one of the outgroup species (lizard) in the previous study (Zhang et al. 2014) (data is not shown). Therefore, in the current study, we used alignments without the gBlocks trimming step, but just highlighted conserved sites with convergent variants of vocal learning birds, which were not trimmed by gBlocks (Table S1).

### Detection of Convergent Variants

We initially developed an algorithm to find amino acid substitutions specific to a group of species, called TAAS analysis (Zhang et al. 2014). It could not detect insertion/deletions (indels) specific to a group of species. In this study, we improved the algorithm to detect indels as well as implemented automated ancestral sequence reconstructions to find convergent variants at amino acid, codon, and nucleotide levels, and named it as ConVarFinder (Fig. S1). ConVarFinder focuses on identifying molecular convergences specific to multiple species from polyphyletic lineages, while TAAS ignored phylogenetic relationships.

First, ConVarFinder identifies mutually exclusive variants either at the amino acid, codon, or nucleotide levels between a target group of species relative to all other species tested. To focus on point mutations, we excluded continuous variants potentially regarded as structural variants. Examples of single amino acid and codon variants were summarized and visualized by using WebLogo (v2.8.2) (Schneider and Stephens 1990). Second, ConVarFinder classifies the mutually exclusive variants into 4 types based on equality or inequality of sequence information in each group: Type 1, mutually exclusive identical amino acid substitutions between Group A and Group B species; Type 2, identical amino acid substitution in Group A and different substitutions in Group B; Type 3, the inverse of Type 2, with different substitutions in Group A, not shared with an identical substitution in Group B; and Type 4, mutually exclusive different sets of amino acid substitutions in Group A and Group B. Third, it infers the evolutionary histories of these substitution variants from their common ancestors to terminal taxa using a given phylogenetic tree. The ancestral sequences were estimated by RAxML (version 8.2.12) (Stamatakis 2014) for codon substitutions with “-f A -m GTRCAT -p 12345” options and for indels converted as binary sequences with “-f A -m BINCAT -p 12345” options. RAxML usually removes the codon sites consisting of all gaps (“—” or “NNN”) in all species, so we trimmed the reduced sequences when we merged the codon and indel sequences to estimate ancestral codon sequences by using a custom Python script (https://github.com/chulbioinfo/ConVarFinder/tree/master/Make_inputs). Based on the outputs of aligned codon sequences and Newick-formatted trees of extant species (terminal nodes) and estimated ancestors (internal nodes), we checked the substitutions on the most recent common ancestral (MRCA = origin) branches of each clade of group A species and classified their evolutionary directions as convergences or divergences. The source codes of ConVarFinder and estimated ancestral sequences are accessible at the following link (https://github.com/chulbioinfo/ConVarFinder).

### Control Sets of Species Combinations From Three Independent Lineages

Considering that we have 6 vocal learning species, we calculated all 6 species combinations of 47 birds in the avian family tree excluding Rifleman, which was 10,737,573 combinations (Fig. S2). Of these, 8,239 combinations of 6 species originated from 3 independent lineages including 3 vocal learning lineages (songbirds, parrots, hummingbirds). From these convergent combinations, we designed two main types of control sets: all 8,238 sets of 6 species with 3 independent origins; and a core control set consisting of 59 possible convergent combinations of species that have a similar phylogenetic history to vocal learners, but contained 6 species originated from 2 clades out of 3 vocal learning clades and 1 vocal nonlearning clade.

### Correlation Tests

To check statistical significances of correlations between various features we discovered in this study, such as, convergences and divergences at the amino acid level (ConSAVs VS DivSAVs), we calculated Spearman rank correlation coefficient as:


$$\mathrm{rho}=\;\frac{\sum_{i}\;(x^{\prime }-m_{x^{\prime }})(y^{\prime }-m_{y^{\prime }})}{\sqrt{\sum_{i}\;{\left(x^{\prime }-m_{x^{\prime }}\right)}^{2}\sum_{i}\;{\left(y^{\prime }-m_{y^{\prime }}\right)}^{2}}}$$

where $x^{\prime }$ and $y^{\prime }$ are each rank of *x* and *y*, respectively, and $m_{x^{\prime }}$ and $m_{y^{\prime }}$ correspond to the means of rank(*x*) and rank(*y*), respectively. By using “cor.test” function with the option method = “spearman” in R package (ver. 3.5.1), we tested correlations between ConSAVs and DivSAVs in the multiple combinations of species (e.g. a set of avian vocal learners, 8,238 all control sets, and 59 core control sets). We then performed linear regression analysis for modeling the relationship between ConSAVs and DivSAVs based on “lm” function, and visualized it with “plot,” “points,” and “abline” function in R package (ver. 3.5.1) (R Core Team 2013). We also performed Bonferroni Outlier Test to check whether the number of convergent variants of vocal learners or other species combinations are outliers, as determined by residuals from the regression model with the “outlierTest” function in R package (ver. 3.5.1) (Fox et al. 2012; R Core Team 2013); option for limitation of the max number of outliers as 3: “n.max = 3.” The source code and dataset to perform above analyses are accessible at the following link (https://github.com/chulbioinfo/ConVarFinder).

### Phylogenetic Features Related to the Number of Molecular Convergences

We performed correlation analyses to determine if there are relationships between convergent variants and various phylogenetic features. Using the branch lengths of the avian total evidence phylogenetic tree from Jarvis et al (Jarvis et al. 2014) (Fig. 1a), we calculated four types of phylogenetic branch measures for convergent groups of species: POB lengths; PTB lengths; distance between terminal branches (DTB); and distance between terminal nodes (DTN; Fig. 2a to d). POB was calculated by multiplying the lengths of the most recent common ancestral (MRCA = origin) branches of each target clade and PTB as branch lengths of terminal taxa. DTB was calculated as a summation of lengths of all branches between the MRCA node of the 47 birds and each terminal taxon, whereas the DTN was calculated as the summation between the MRCA node and the most recent ancestral nodes of each terminal taxon (Fig. 2a). The source code to calculate each phylogenetic feature is accessible at the following link (https://github.com/chulbioinfo/ConVarFinder).

### PCA and maximum likelihood Tree Analyses for Rifleman

Principle component analysis (PCA) was performed using the method as implemented in JalView (Waterhouse et al. 2009). Focusing on the 148 avian vocal learner–specific SAV sites (ConSAVs + DivSAVs) and 24 avian vocal learner–specific ConSAV sites alone, pair-wise scores between bird species was computed by summing the substitution scores from BLOSUM62. Then, we performed spectral decomposition of the score matrix to obtain principal component vectors and eigenvalues of the respective vectors. The PCA biplot was computed using PC1 and PC2 vectors. For the maximum likelihood tree, we constructed it using MEGA (Kumar et al. 2018), and selected the JTT model, on the part of the amino acid sequence alignment of all 148 vocal learner–specific SAV or 24 vocal learner–specific ConSAV sites.

### GO Functional Annotations and Gene Network Analyses

We conducted GO analysis by using g:Profiler (v 0.3.5.) (Raudvere et al. 2019) with the default option and ClueGO (ver. 2.3.3.) in Cytoscape (Shannon et al. 2003) with the following options: GO BiologicalProcess-GOA (released in 08.04.2016); all of GO tree interval; all of GO Term/Pathway selection; multiple testing correction was done by Bonferroni analyses (adjusted *P*-value < 0.05); and default options of others. We then tested whether the number of genes is correlated with the number of significant GO terms, by applying regression analyses using “lm” function. We visualized the results with “plot,” “points,” and “abline” functions in the R package (ver. 3.5.1) (R Core Team 2013).

We then focused on two GO lists enriched for learning processes: vocal learner–specific ConSAV gene list and a control DivSCV and DivSNV gene lists (different codon convergences specific to Dalmatian pelican, little egret, houbara bustard, red-crested turaco, white-throated tinamou, and ostrich). We searched for networks between the enriched genes for learning by analyzing protein-protein interactions among convergent genes, using CluePedia ver. 1.3.3. (Bindea et al. 2009) in Cytoscape (Shannon et al. 2003), selecting the following databases: STRING-ACTIONS_v10.0 (released in 07.05.2015); activation v10.0; binding v10.0; catalysis v10.0; expression v10.0; inhibition v10.0; ptmod v10.0, and reaction v10.0. Sequences of the convergent variants of gene lists of vocal learners and a control set associated with learning were visualized by WebLogo (v2.8.2) (Schneider and Stephens 1990; Crooks et al. 2004).

### Fixed Differences of Vocal Learner–Specific Amino Acid Variants Within Populations of Zebra Finch and Chicken

More than 20 million (20,739,045) and 1.6 million (1,661,545) variants have been reported in chicken (*n* = 9,586) and zebra finch (*n* = 1,257), respectively, according to Ensembl database release 84 (Yates et al. 2016; Zerbino et al. 2018). Hence, we performed additional analysis to check if the vocal learner–specific ConSAV and DivSAV sequences we identified were not due to within species variation. Local alignment was conducted for the protein-coding sequences (CDSs) containing these vocal learner ConSAVs using BLAST (ver. 2.8.1) (Madden 2004) to find the position of the SAVs on the chromosome sequence of chicken (Galgal4) and zebra finch (taeGut3.2.4) according to Ensembl database release 84. Fixation of sequence in a species was assessed by comparing the nucleotide site in question in an alignment from Ensembl dbSNP build 145 and 139 of chicken and zebra finch, respectively (Sherry et al. 2001).

We also performed additional fixation analyses on several genes amplified by PCR from red blood cells of zebra finch (*n* = 3 males and 3 females) and chicken (*n* = 3 males and 3 females). The *DRD1B* gene was cloned from genomic DNA by using zebra finch specific primers (forward 5′-GCC CTG CGT CAG TGA GAC CA-3′ and reverse 5′-CCG CCA GCC CCC TGT ATG AC-3′) and white-leghorn chicken specific primers (forward 5′-CAG ATC TCC CCC GAC CCC GA-3′ and reverse 5′-GGC AAC AAT GCC GCC TGG AG-3′). The PCR reaction was conducted in a total volume of 20 μL containing 100 ng genomic DNA, 10× PCR buffer, 0.4 μL dNTP (10 mM each), 10 pmol of each primer, and 0.5 U Taq polymerase (BioFACT) in the following thermocycling conditions: 2 min at 95 °C, followed by 35 cycles of 20 s at 95 °C, 40 s at 60 °C, 2 min at 72 °C, and, finally, 5 min at 72 °C. The PCR products were cloned into the pGEM-T easy vector (Promega) and sequenced using an ABI Prism 3730 XL DNA Analyzer (Thermo Fisher–Applied Biosystems).

### Positive Selection on MRCA Branches of Vocal Learners and the Closest Control Set

The rate of nonsynonymous substitutions (dN), the rate of synonymous substitutions (dS), and *ω* = dN/dS were estimated along each branch of the phylogenetic tree and across sites by using the branch-site model A, implemented in codeml within PAML ver. 4.6 (Yang 2007) with F3X4 codon frequencies. We assumed the vocal learning trait independently originated from the most recent common ancestor branch of each vocal learning lineage (Cahill et al. 2021). Log LRT (*D*-value) was performed to compare the null hypothesis with a fixed *ω* (model 2) for neutral and negative selection (*ω* ≤ 1) and an alternative hypothesis with an estimated *ω* (model 2) for positive selection (*ω* > 1). Orthologs with *ω*2 Foreground >1 and number of accelerated sites (Bayes empirical Bayes > 0.5) > 0 were retained (branches tested for positive selection are referred to as “foreground” branches and all other are referred to as “background” branches). The data set of codon sequences of each gene list, including alignment gaps in species, was analyzed with a codeml option (cleandata = 0) and robust cutoff of adjusted *P*-value (<0.05; FDR). FDR was calculated in R (ver.3.0.1). Genes with three types of SAVs (all, convergence, and divergence) were analyzed with consideration on likelihoods and statistical significances of their alternative models for positive selection (*D* > 1 and adjusted *P*-value <0.05, respectively) specific to vocal learner set and the closest control set (3 songbirds, 2 parrots, and the closest vocal nonlearning relative species of hummingbirds, swift).

### Meta-Analyses for Vocal Learning Candidate Genes

We collected six published gene interaction or expression data sets associated with vocal learning for three major types of comparisons: *FOXP2* targets, singing-induced genes in song nuclei, and differentially expressed genes in song nuclei relative to their surrounding nonvocal motor brain regions. Out of the 8,295 singleton orthologous gene set, 83.6% (6,932 genes) was in at least one of data sets with same gene symbols.

1. *FOXP2* targets: A data set of 786 target genes of *FOXP2*, the foremost language associated gene, in the ZEBrA database (Lovell et al. 2020) and ChEA database (Lachmann et al. 2010).
2. Singing-regulated genes: A data set of 165 singing-regulated genes in various song nuclei, in the ZEBrA database (Lovell et al. 2020). A data set of 2,013 singing-regulated genes in Area X (Hilliard et al. 2012a). A data set of 852 singing-regulated genes in Area X compared with the striatum ventral to Area X (VSP) which were significantly enriched in functional annotations (Hilliard et al. 2012b). A data set of 2,740 singing-regulated transcripts in HVC, RA, LMAN, and Area X song nuclei (Whitney et al. 2014).
3. Specialized expressed genes: A data set of 2,640 genes with specialized up- or downregulated expression in among song nuclei HVC, RA, and Area X compared with their surrounding nonvocal brain regions (Lovell et al. 2018). A data set of 5,473 genes with specialized expression in 4 song nuclei, HVC, RA, LMAN, and Area X relative to their surrounding nonvocal motor brain regions (Gedman et al. 2022).

Based on the above data sets of candidate genes for vocal learning, we calculated their proportions in lists of genes with single amino acid evolution that we detected in this study.

### Amino Acid Patterns of Candidate Convergent Sites of Vocal Learners in 363 Avian Genome Alignments

For 6 key candidate genes (*B3GNT2, DRD1B, FNDC1, PIK3R4, PRKAR2B,* and *SMPD3*) and 2 more learning-related genes (*LRRN4* and *TANC1*), we analyzed the reference-free genome-wide CACTUS alignment of 363 bird species generated by the second phase of Avian phylogenomics consortium (B10K) (Feng et al. 2020). Among the 363 species, there was no vocal learning outlier species such as, *P. nudicollis* (Kroodsma et al. 2013) among the 26 suboscines and *B. lobata* (Ten Cate and Fullagar 2021) among the 7 Anseriformes. To find genomic positions of vocal learner–specific ConSAVs of candidate genes, we parsed out the zebra finch's (taeGut2 assembly: GCF_000151805.1) coding sequences of codons that produce the amino acid convergence in question, with 10 bp up- and downstream of in coding gene alignments of 1:1 orthologs. Next, we manually scanned the alignment and genomic positions of the 21 bp parsed regions for any errors (Table S3) by using the UCSC genome browser (Nassar et al. 2023). Based on the zebra finch's genomic locations of vocal learner–specific ConSAVs of candidate genes, we extracted chromosome-wide MAF files by setting zebra finch as the reference species from the published HAL file (https://cgl.gi.ucsc.edu/data/cactus/363-avian-2020.hal) (Feng et al. 2020) by using hal2maf in the HAL tool (Hickey et al. 2013). We then extracted alignment blocks with vocal learner–specific ConSAVs, changed the species order following the 363 species avian family tree (https://cgl.gi.ucsc.edu/data/cactus/363-avian-2020-phast.nh) (Feng et al. 2020), and finally extracted codon sequences of all species at the vocal learner–specific ConSAV sites of candidate genes in the alignment blocks by using the in-house code, MafScan.py (https://github.com/chulbioinfo/CSAVanalysis).

At each vocal learner–specific ConSAV site, we assessed amino acid enrichments in vocal learning clades (*n* = 154) and the remaining vocal nonlearning clades (*n* = 209) by using sequence Logo (Crooks et al. 2004) and calculated. We display the proportions of vocal learner–type amino acid patterns in a total 22 clades using Microsoft Excel and Adobe Illustrator.

### Protein Functional Domain Analysis of Vocal Learner–Specific Amino Acid Variants

We analyzed 125 peptide sequences that existed in zebra finch within 135 genes, which contained 148 ConSAVs in vocal learners. We conducted functional domain analyses on these peptide sequences using the NCBI conserved domain search tool (Marchler-Bauer et al. 2017; Wang et al. 2023). For two key candidate genes, *B3GNT2* and *DRD1B*, we examined their protein structures as predicted by AlphaFold2 (Jumper et al. 2021), accessing data through their database (https://alphafold.ebi.ac.uk/). To address mismatches in peptide sequence versions, we manually scanned and annotated the homologous sequences at the ConSAV sites.

### Institutional Review for Animal Cares and Experiments

The care and experimental use of animals (zebra finch or chicks) were approved by the Institute of Laboratory Animal Resources, Seoul National University (SNU-150827-1) and the Rockefeller University IACUC. The experimental animals were maintained according to a standard management program at the University Animal Farm, Seoul National University or the Rockefeller University. The procedures for animal management adhered to the standard operating protocols of the laboratory at Seoul National University, Korea, or at the Rockefeller University, USA.

## Supplementary Material

evaf112_Supplementary_Data

## Data Availability

All codes and datasets required to reproduce the results are available at https://github.com/chulbioinfo/ConVarFinder.
