# Supplementary material for: Insights into Convergent Evolution From Studying Amino Acid Patterns in Independent Lineages of Birds
Source: Genome Biol Evol. 2025 Dec 15;17(12):evaf112. doi: 10.1093/gbe/evaf112 (PMC12703197; doi:10.1093/gbe/evaf112)
Supplement: evaf112_Supplementary_Data [file evaf112_supplementary_data.zip › GBE_240222.R1_SupplementaryInformation_Clean.docx]

**Supplementary Information**

**Insights into Convergent Evolution from Studying Amino Acid Patterns in Independent Lineages of Birds**

Lee et al.

**Contents**

Supplementary data legends

Supplementary figures

**Supplementary data legends**

**Supplementary table S1. Vocal learner specific single amino acid variants in 47 bird species.** (**Column A**) orthologous gene set ID. (**Column B**) Gene symbol. (**Columns C-H**) Vocal learner-specific single amino acid variants (AVL-SAV sites) and their detailed types classified with amino acid patterns at terminal nodes. (**Column I-BC**) Amino acid profiles of 47 bird species. (**Columns BD,** **BE**) Node IDs in internal branches from most recent common ancestors of each vocal learning clade to terminal nodes. (**Columns BF-BI**) Evolutionary directions of amino acid variants from the most recent common ancestors of each vocal learning clade to terminal nodes. (**Columns BJ-BO**) Vocal learner-specific single codon variants (AVL-SCV sites) that cause AVL-SAVs and their detailed types classified with codon patterns at terminal nodes. (**BP-DJ**) Codon profiles of 47 bird species. (**Columns DK-DN**) Evolutionary paths of codon variants from most recent common ancestors of each vocal learning clade to terminal nodes. (**Columns DO-ER**) Vocal learner-specific single nucleotide variants (AVL-SNV sites) causing AVL-SAVs and their detailed types and evolutionary directions. (**Columns ES-EV**) Positive selection signatures in AVL-SAV sites. (**Column EW**) AVL-SAVs on conserved regions which were not trimmed by Gblocks.

**Supplementary table S2. Gene data sets for vocal learning birds.** (**Column A**) Orthologous gene set ID. (**Column B**) Gene symbol. (**Columns C-T**) Genes with amino acid variants under positive selection specific to vocal learning clades and the closest control set. AVL: Avian vocal learners (3 songbirds, 2 parrots, and 1 hummingbird), Swift: the closest control set (3 songbirds, 2 parrots, and 1 swift), SAV: single amino acid variants, ConSAV: Convergent SAV, DivSAV: Divergent SAV, PS: positive selection, *D*: Likelihood ratio value for positive selection, and Adj. *p*: *p*-value adjusted by FDR. (**Columns U-AB**) Meta data sets of precedent candidate genes related to vocal learning. Matching_Symbols_MetaDataSets (>=1): genes with same symbols between singleton orthologs and at least one of the meta data sets, ‘FOXP2_target (ZEBrA)’ and ‘Singing (ZEBrA)’ from the ZEBrA database [(Lovell et al. 2020)](https://paperpile.com/c/n4VSSQ/FYZN), ‘FOXP2_target (ChEA)’ from ChEA chip-seq databse [(Lachmann et al. 2010)](https://paperpile.com/c/n4VSSQ/4x1P), ‘Singing_AreaX (Hilliard_2012)’ [(Hilliard, Miller, Fraley, et al. 2012)](https://paperpile.com/c/n4VSSQ/REfw), ‘Singing_AreaX_vs_VSP (Hilliard_2012)’ [(Hilliard, Miller, Horvath, et al. 2012)](https://paperpile.com/c/n4VSSQ/6d8k), ‘Singing (Whitney_2014)’ [(Whitney et al. 2014)](https://paperpile.com/c/n4VSSQ/LL41), ‘DEG_songnucleiVSsurrounding (Lovell_2018)’ [(Lovell et al. 2018)](https://paperpile.com/c/n4VSSQ/IY5m), and ‘DEG_songnucleiVSsurrounding (Gedman_2022)’ [(Gedman et al. 2022)](https://paperpile.com/c/n4VSSQ/83y4).

**Supplementary table S3. Genic and genomic locations in zebra finch of candidate sites with amino acid convergences of vocal learning birds.** Shown are six genes containing a vocal learner ConSAV site under positive selection present in at least one other gene data set associated with vocal learning behavior or circuity. The genomic positions are from the zebra taeGut2 finch assembly (GCF_000151805.1). Included are 20 bp (±10bp) around the ConSAV site.

**Supplementary table S4. Amino acid profiles at candidate sites specific to avian vocal learners among 363 avian species.** Shown are sites for six vocal learner ConSAV genes under positive selection supported by at least one of other gene data sets for vocal learning. Amino acid profiles at candidate sites were extracted from the CACTUS alignments of 363 bird species. ‘B10K phase 1’: same species or relative in the 1st phase of B10K genomes of 48 birds. Red labelled amino acid, are those found in the vocal learning birds among the 48 species.

**Supplementary table S5. Conserved functional domains with vocal learner-specific single amino acid variants.** (**Column A**) gene symbol. (**Column B**) peptide positions with vocal learner-specific single amino acid variants (SAVs). (**Column C**) conserved domain database (CDD) hit type. (**Column D**) domain model's position-specific scoring matrix (PSSM) ID. (**Column E,F**) peptide position of start and end of the estimated domain. (**Column G,H**) E-value and bitscore of NCBI conserved domain search. (**Column I-L**) detailed information of functional domain.

**Supplementary figure legends**

**Supplementary fig. S1. Flow chart of convergent variant finder (ConVarFinder).** Process to find convergent single seqeuence variants at the amino acid, codon, and nucleotide levels (ConSAV, and ConSCV, and ConSNV, respectively). Ancestral sequences were estimated using RAxML [(Stamatakis 2014)](https://paperpile.com/c/n4VSSQ/fuyNF). Stadiums indicate beginning and ending of the program. Rectangles indicate sets of operations. Rhomboids indicate input/output. Blue boxes, algorithmic steps used; grey boxes, needed input.

**Supplementary fig. S2. Flow chart to design control sets of species combinations.** Process to calculate combinations of Stadiums indicate beginning and ending of the process. Rectangles indicate a set of operation. Rectangle with a wavy base indicates a species list. Rhomboids indicate input/output. Grey boxes, needed input.

**Supplementary fig. S3. Correlation analyses among various convergent evolution, molecular, and phylogenetic parameters for the core control set of species combinations.** Histograms on the diagonal of the matrix are of frequencies of each convergent variant and values of each phylogenetic feature visualized, and list all the names of the variables compared. Graphs along the lower left of the matrix are regression plots; grey, orange, and red spots indicate all control sets (n=57), the closest control set of vocal learners (n=1), and the set of avian vocal learners (n=1), respectively. The statistical values for these regression graphs are in upper diagonal matrix, *p* values and Adjusted *R*^2^ (*p*<0.05*, *p*<0.01**, and *p*<0.001***). Black lines and black ‘X’ marks indicate regression lines and outliers, respectively. POB = product of origin branch lengths, PTB = product of terminal branch lengths, DTB = distance between terminal branches, DTN = distance between terminal nodes, SAV = convergent + divergent single amino acid variants, ConSAV = convergent SAV, DivSAV = divergent SAV, SCV = single codon variants, ConSCV = convergent SCV, DivSCV = divergent SCV, SNV = convergent + divergent single nucleotide variants, ConSNV = convergent SNV, DivSNV = divergent SNV. Correlations of core control sets are shown in **Supplementary fig. S4**.

**Supplementary fig. S4. Examples of fixed and non-fixed differences within each population.** The central table indicates convergent single amino acid variants of vocal learning birds in *DRD1B* and *OTOA*. Numbers in parentheses indicate positions in peptide alignments of each gene. Bold characters in the species name column indicate representative species of vocal learners and non-learners. Amino acid and codon columns show amino acids and codons of each species at the vocal learner SAV sites of each gene. Blank and ‘- (gap)‘ indicates absence of orthologous gene in the species’ genome and deletions in the species. The SAV site in the *OTOA* gene of chicken indicates a nonsynonymous SNP in the chicken population (dbSNP149, number of samples = 9,586), highlighted in grey. Except for the case of *OTOA* gene of chicken, all of vocal learner SAV sites are conserved within zebra finch population (dbSNP139, number of samples = 1,257) and the chicken population.

**Supplementary fig. S5. Fixed differences of the avian vocal learner-specific amino acid convergences in *DRD1B.*** Shown are sequences determined from PCR reactions from individual animals. All 3 male and 3 female zebra finch samples showed fixation of the vocal learner-type codon (GCC) and all 3 male and 3 female chicken samples showed the vocal non-learner-type codon (GTC) at the ConSAV site.

**Supplementary fig. S6. Rifleman’s amino acid profile. a, b**) Principle component analysis (PCA) of 148 vocal learner-specific SAV sites and 24 vocal learner-specific ConSAV sites among all species examined, respectively. **c, d**) Consensus trees of 148 vocal learner-specific SAV sites and 24 vocal learner-specific ConSAV sites among all species examined, respecitvely. Red, avian vocal learners; Grey, avian vocal non-learners; Purple, rifleman.

**Supplementary fig. S7. Learning related gene network of four convergent genes in avian vocal learning species**. Red hexagons indicate the four vocal learner ConSAV genes that function in learning. Transparent red hexagons indicate muti-cellular location of the candidate genes. Out of 4 learning-associated genes, *DRD1B* and *PRKAR2B* interact with cAMP, and/or its target *CREB1*, a transcription factor well known for its role in converting short-term memories into long-term memories. Blue rectangles indicate traits related to learning. Black arrows and dashed blue arrows indicate trait-gene relationships and trait-trait relationships, respectively.

**Supplementary fig. S8. Meta-analysis for SAV genes of vocal learners and their closest control set with swift.** Right column, sources and type of gene sets associated with vocal learning behavior or circuits in birds. Remaining columns, hypergeometric correlation values of overlap of the prior published gene sets with three types of single amino acid variants (SAVs, ConSAVs, and DivSAVs) in the avian vocal learner set (songbirds, parrots, and hummingbird) or its closest control set (songbirds, parrots, and swift). Each analyses has 2 sub-sets by considering statistical significances of positive selection (likelihood ratio test and chi-square test with multiple testing correction using FDR). The prior gene data sets are of 3 major types: targets of *FOXP2*; singing regulated genes in zebra finch song nuclei; and differentially expressed genes of song nuclei compared to their surrounding non-vocal regions. (see more details in **Materials and Methods**).

**Supplementary figures**

**
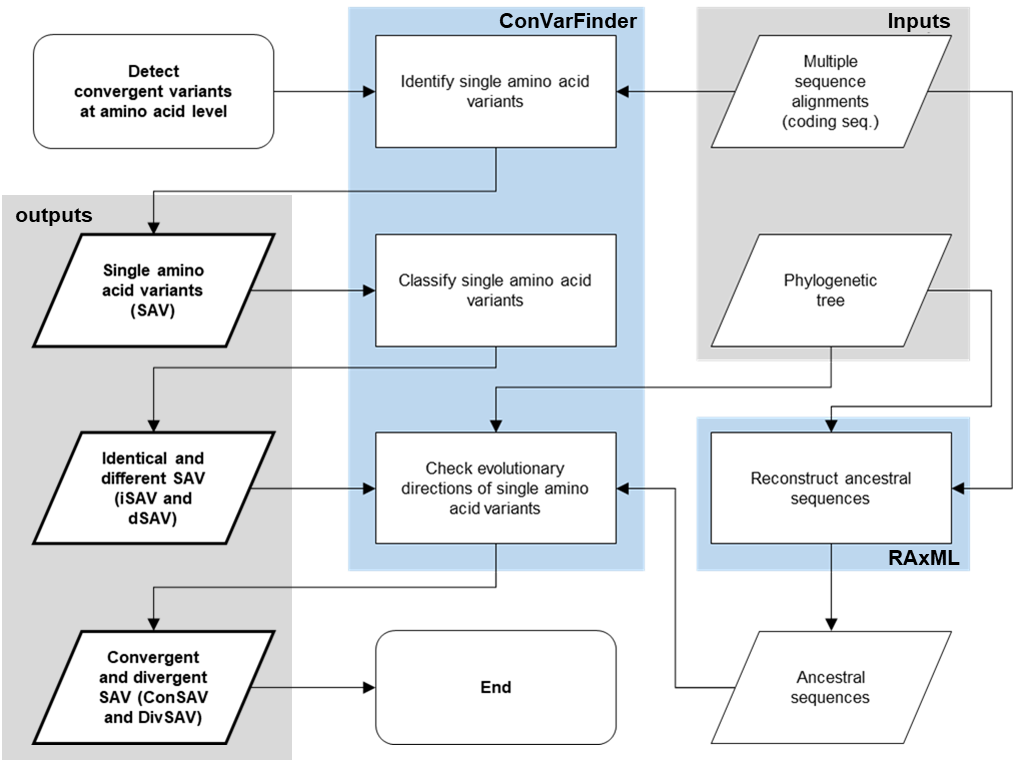
**

**Supplementary fig. S1.**

**
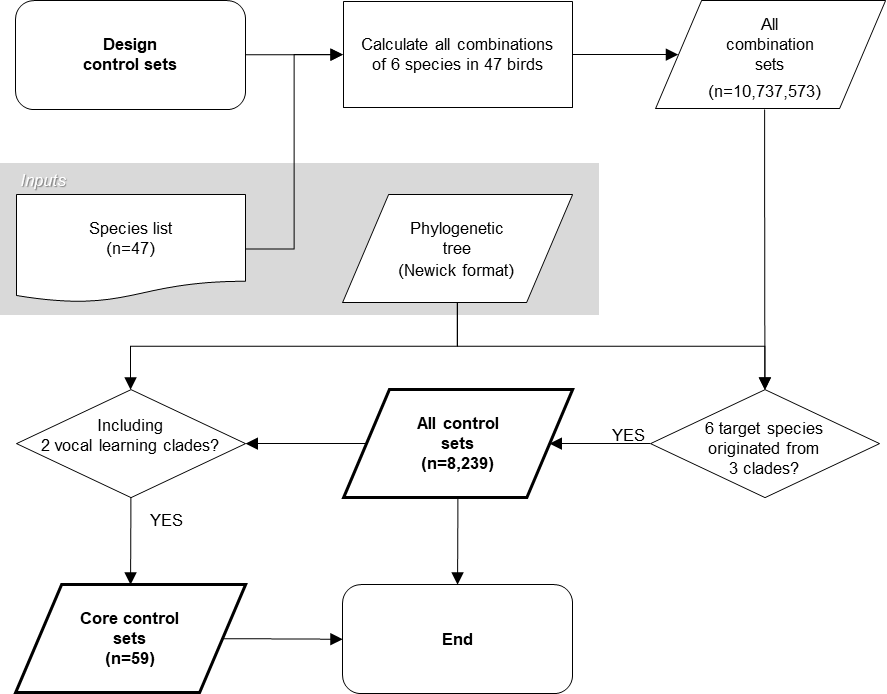
**

**Supplementary fig. S2.**

**
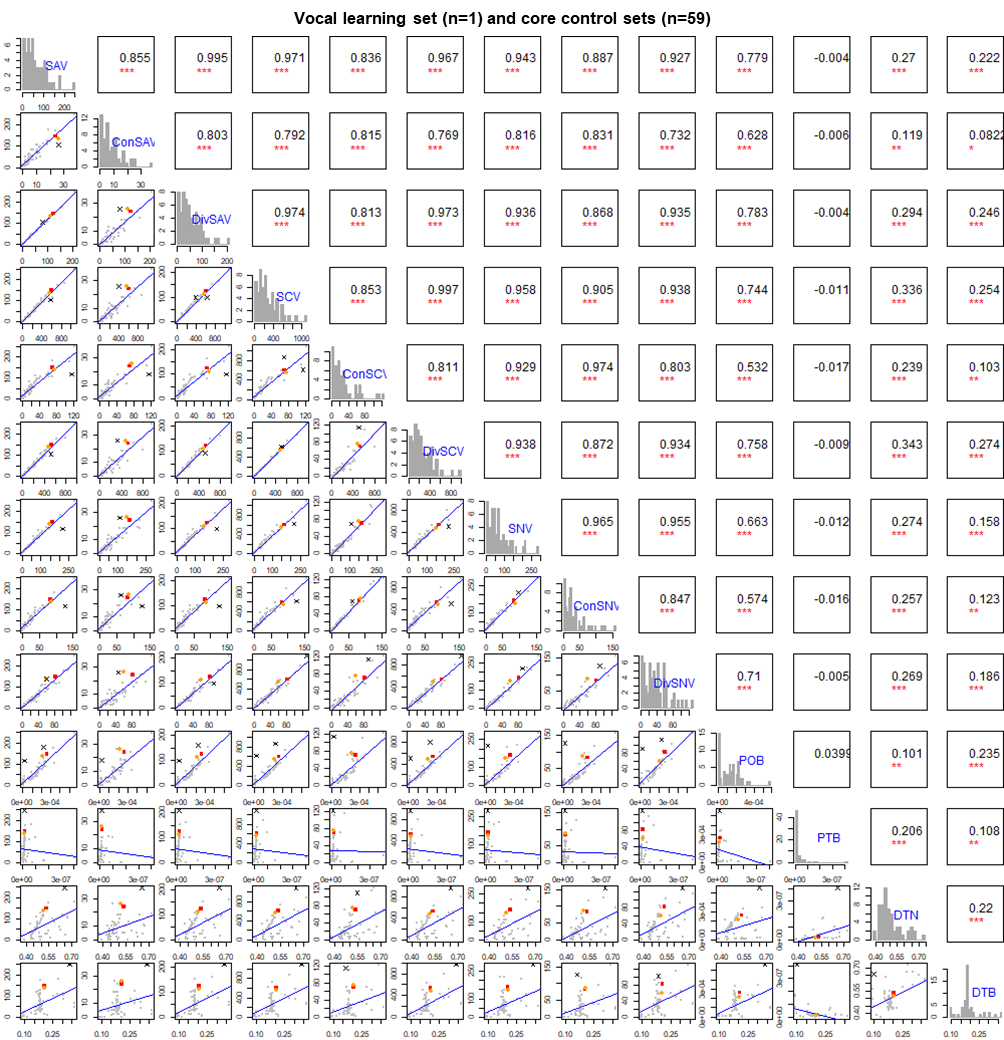
**

**Supplementary fig. S3.**

**
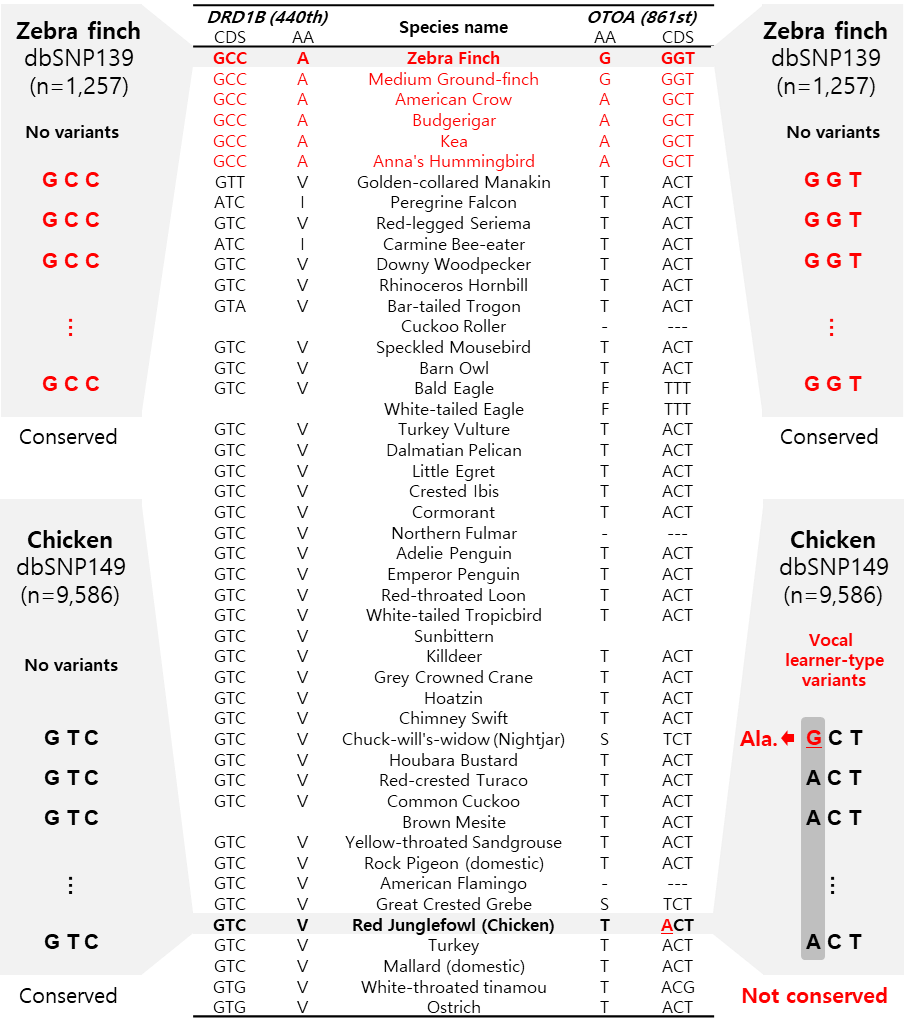
**

**Supplementary fig. S4.**

**
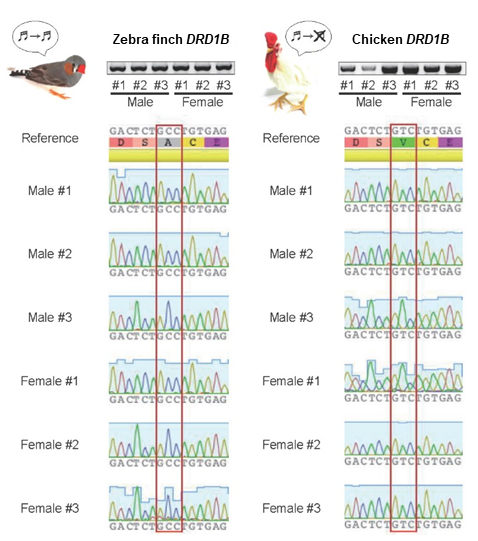
**

**Supplementary fig. S5.**

**
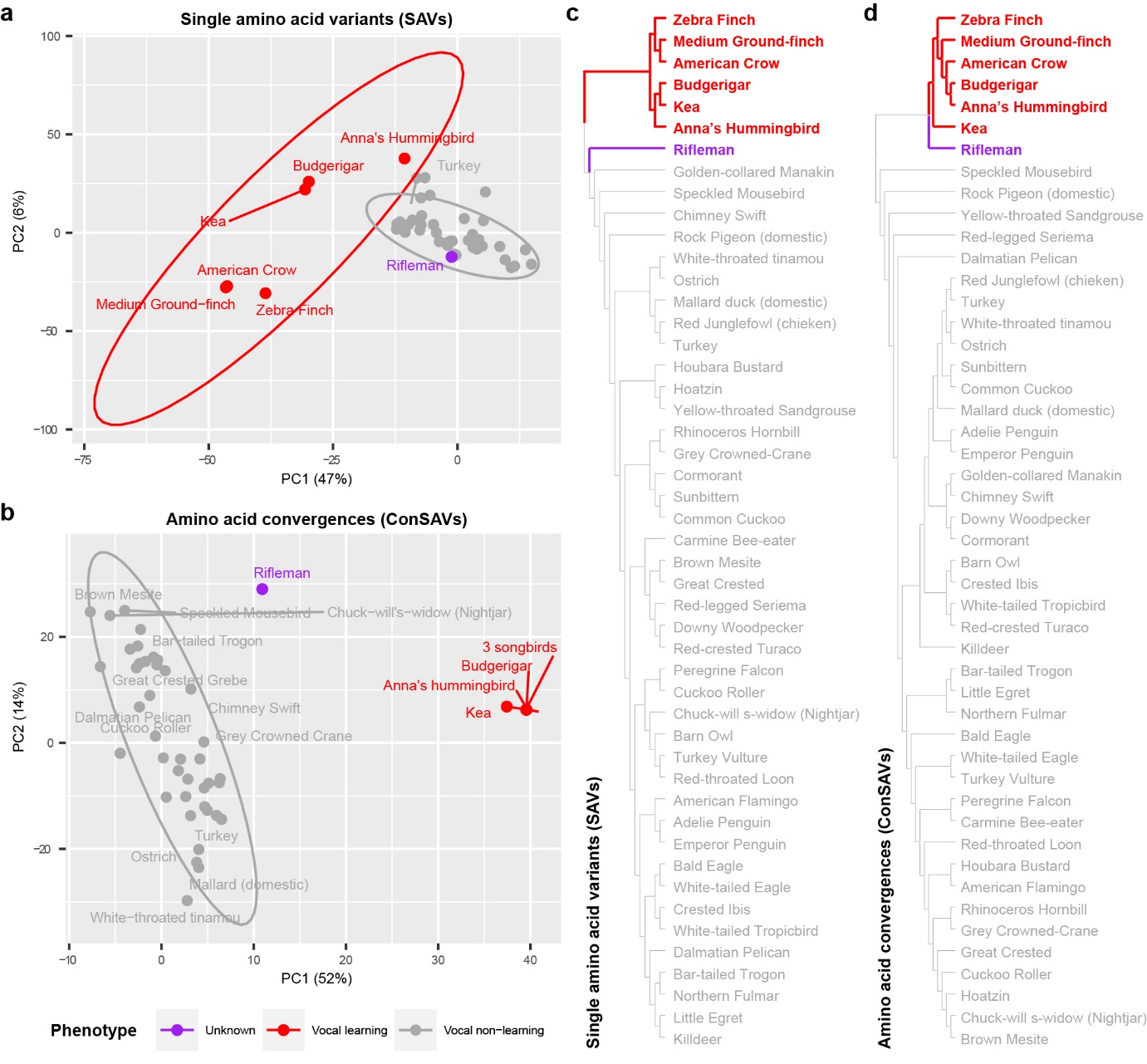
**

**Supplementary fig. S6.**

**
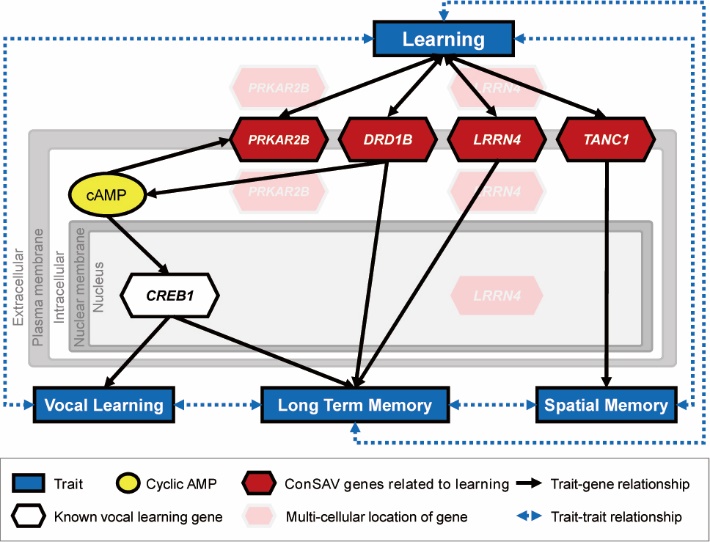
**

**Supplementary fig. S7.**

**
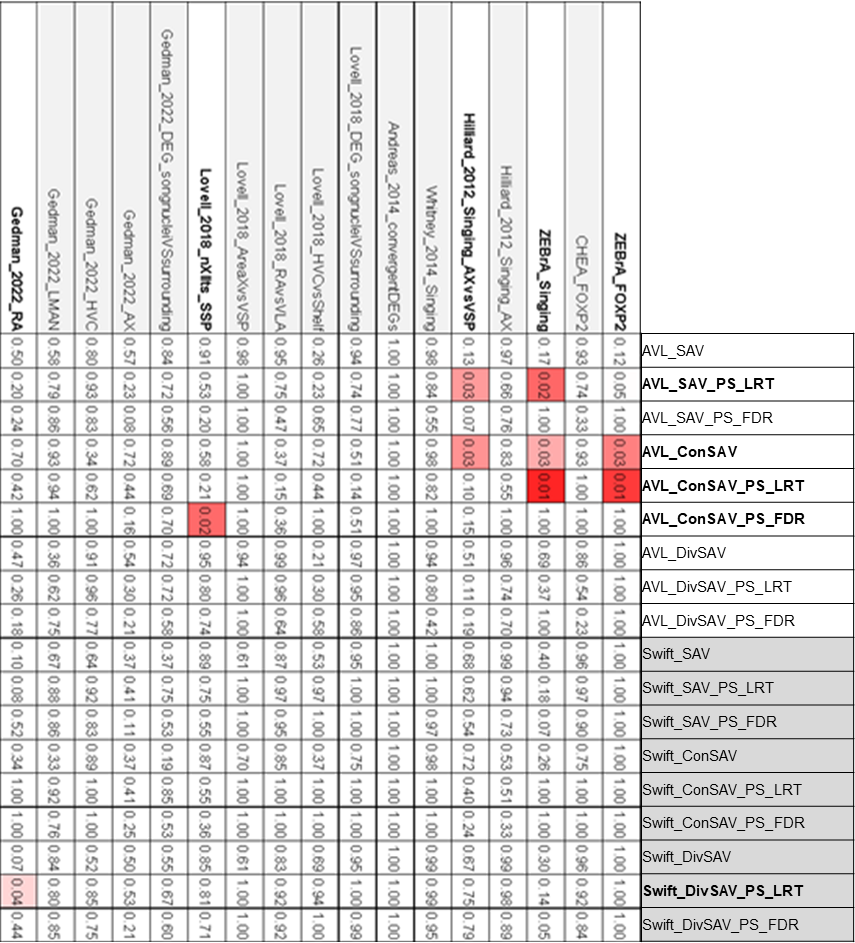
**

**Supplementary fig. S8.**
